# Supplementary material for: Ocean acidification at the Toarcian Anoxic Event captured by boron isotopes in the lime mud record
Source: Commun Earth Environ. 2025 Jul 5;6(1):533. doi: 10.1038/s43247-025-02510-y (PMC12228580; doi:10.1038/s43247-025-02510-y)
Supplement: Supplementary file 2 — Supplementary Information [file 43247_2025_2510_MOESM2_ESM.pdf]

Supplementary Materials for

Ocean acidification at the Toarcian Anoxic Event captured by boron isotopes in the lime mud record

Simone A. Kasemann[1]*, Tina Klein[1], Richard A. Boyle[2], Clemens V. Ullmann[3], Martin Aberhan[4], Anette Meixner[1], Luís V. Duarte[5], Timothy M. Lenton[2], Veronica Piazza[4], Rachel A. Wood[6]

Affiliations

[1]Faculty of Geosciences and MARUM – Center for Marine Environmental Sciences, University of Bremen, 28359 Bremen, Germany.

[2]Global Systems Institute, University of Exeter, Exeter EX4 4QE, United Kingdom.

[3]Camborne School of Mines & Environment and Sustainability Institute, University of Exeter, Penryn TR10 9FE, United Kingdom.

[4]Museum für Naturkunde, Leibniz Institute for Evolution and Biodiversity Science, 10115 Berlin, Germany.

[5]University of Coimbra & MARE, 3030-790 Coimbra, Portugal.

[6]School of GeoSciences, University of Edinburgh, Edinburgh EH9 3FE, United Kingdom.

*Correspondence to: kasemann@uni-bremen.de

This PDF file includes:

Supplementary Note 1 and 2

Supplementary Discussion

Supplementary Methods

Supplementary Figures 1 to 9

Supplementary Tables 1 to 7

Supplementary References

Supplementary Note 1

Rabaçal/Fonte Coberta - Lusitanian Basin (Portugal)

The origin of the Peri-Tethyan Lusitanian Basin is related to the opening of the Atlantic Ocean during the Mesozoic[1-3]. It comprises a small, shallow (less than 160 to 200 m depth[4]) and narrow north to south elongated basin that was limited to the east by the Iberian Massif, to the north by shallow seas and to the west by emerged horst blocks. To the south, it was open to the epicontinental basins of the NW Tethyan realm[5] (Fig. 1). Located in the Lusitanian Basin are the Rabaçal (40°03'08.0"N 8°27'30.5"W) and Fonte Coberta (40°03'36.5"N 8°27'33.4"W) composite section, approximately 1 km south of the small village Fonte Coberta and 2.5 km north of the village Rabaçal. This section represents a former homoclinal northeast dipping outer/mid-ramp environment during the Early Jurassic with a paleo-water depth below 200 m[6-8]. The depositional environment is thought to have remained below storm-wave base throughout the studied time interval[6]. The biozonation in this succession is well defined by ammonites, nannofossils and dinoflagellate cysts[9-12].

The studied part of this section encompasses 73 m, from which 17 m are Pliensbachian (Margaritatus and Spinatum Zones – for an overview of the biostratigraphy see Supplementary Figure 1) and 55 m are early to middle Toarcian (Polymorphum, Levisoni and Bifrons Zones) in age (Fig. 2). Lithologically, the studied section belongs to the Lemede Formation (Pliensbachian) that spans across the Margaritatus and Spinatum Zones and consists of centimetre scale marl and decimetre scale bioturbated limestone alternations[7], and three informal members of the São Gião Formation (Toarcian) (Fig. 2)[e.g.13 and references therein]. The marly limestone with Leptaena fauna (MLLF) member (mb) is the base of the São Gião Formation. It comprises an alternation of bioturbated greyish decimetre-thick marls with centimetre-thick fossiliferous micrites to biomicrites / wackestones. At the top of this member, a thick limestone bed occurs (at approximately 6 m height in the lithological log, Fig. 2), which serves as a reference level for the Lusitanian Basin, with exception of the southern sectors. The thin nodular limestone (TNL) member commences at about the Polymorphum - Levisoni zone boundary, marking the onset of the T-OAE. The TNL mb is identified by a distinct change in lithology that can be followed throughout the Lusitanian Basin: few cm-thin marlstones alternated with brownish claystone and micritic to microsparitic limestones. The succession appears nodular and has a typical total thickness of about 7 m. The marls and marly limestones with *Hildaites* and *Hildoceras* (MMLHH) mb span from the middle Levisoni Zone up to the upper Bifrons Zone and return to decimetre and metre-thick marl and marly limestone alternations. Remarkably, the typical black shales, characteristic for the T-OAE elsewhere, are not developed in this section. In addition to the lack of black shales, total organic carbon levels are rather low[14] and trace fossils indicate oxygenated bottom conditions[15, 16, 17].

Barranco de la Cañada - Iberian Basin (Spain)

The Barranco de la Cañada section is located in the former Iberian Basin (Fig. 1), which is situated in the Iberian Range, representing a moderately deformed intraplate mountain chain

(Celtiberian Chain) that was formed by a NW trending fold and thrust belt[18]. During the Early Jurassic, sedimentation was dominated by shallow carbonate platforms and transgressive hemipelagic alternations of limestone and marls deposited in an intracontinental basin during a post-rift episode[19, 20]. The outcrop is located about 5 km west of the village Albarracín (40°23'53.4"N 1°30'07.4"W). Its complete succession from the upper Pliensbachian to middle Toarcian makes it well suited to investigate the T-OAE.

The studied interval spans 48 m, from which 15 m cover the Pliensbachian (Barahona Formation) and 33 m belong to the Toarcian (Turmiel Formation), reaching up to the Bifrons Zone (Fig. 2). Facies, ammonite zones and faunal composition are described in detail[16, 21]. The succession represents a former, gently sloping homoclinal mid-ramp environment[16, 22] with a paleo-water depth of about 40 to 70 m. Compared to the Rabaçal/Fonte Coberta section, the Barranco de la Cañada section was deposited in a slightly shallower water setting and less influenced by tectonic activity, but both sections were most of the time below storm wave base[23, 24].

The lithology in this section is an alternation of marls and limestones. The Pliensbachian Margaritatus and lower Spinatum zones are dominated by metre-thick limestones with frequently occurring biomicrites. Up to the Pl-To boundary, limestones (biomicrites) occur in decimetre-thick layers alternating with marly limestones and calcareous marls. The whole Tenuicostatum Zone (corresponding to the Polymorphum Zone in the Lusitanian Basin) is dominated by marls with a few centimeter-thick marly limestone layers. The subsequent Serpentinum Zone (corresponding to the Levisoni Zone in the Lusitanian Basin) as well as the Bifrons Zone are made up of an alternation of centimeter-tick limestones varying in their clay content and marls[16]. In line with the findings in the Lusitanian Basin, this area of the Iberian Basin also appeared to be oxygenated during the T-OAE interval, as suggested by the absence of black shales, low total organic carbon content, and abundant body fossils and ichnofossils[16, 25 and references therein].

Supplementary Note 2

<u>Age Model</u>

The age model for this study is based on the latest findings on the biozonations published for Rabaçal/Fonte Coberta[11, 12, 26], and for the Barranco de la Cañada section[6, 16], and by observations in the field. The absolute ages for the ammonite zones are defined by the Geological Time Scale 2016[27] and given in the Supplementary Figure 1. The age model was established with the assumption that sedimentation rates within each ammonite zone were constant throughout the studied interval.

Supplementary Discussion

<u>Detrital component XRD Analyses</u>

Detrital components of the carbonate rock, especially clay minerals, may contaminate the original elemental mass fraction and isotopic composition of the carbonate during dissolution of the sample. So far, the dissolution techniques applied[28, 29] avoided any detectable contamination by the detrital clay fraction. The low Al mass fractions (< 116 ug g^{-1}, Supplementary Table 4) in our sample solutions is supporting this observation. The estimated amount of the detrital fraction for Rabaçal/Fonte Coberta (Portugal) and Barranco de la Cañada (Spain) ranges from 2 to 22 wt.%, and is on average 14 and 10 wt.%, respectively. To assess the amount and composition of the detrital component in the micritic carbonate samples, X-Ray Diffraction (XRD) analyses were performed with an X'Pert Pro multipurpose diffractometer (research group Crystallography, University of Bremen). The majority of the detrital fraction comprises quartz and mica; the clay mineral content is commonly below 9 % (Supplementary Table 5).

<u>Environmental controls on the $\delta^{11}B$ composition of micrite</u>

<u>Temperature:</u> Temperature reconstructions, which are based on a negative shift of 1.00 ± 0.09 ‰ in $\delta^{18}O$ values from low-Mg calcite brachiopods at Spain, revealed a rise in seawater temperatures of 3.5 ± 0.3 °C at the seafloor of the shallow Iberian Basin during the T-OAE[25]. Since the dissociation constant of boric acid (pK_B) decreases with increasing temperature[30], the computed rise in seawater temperature at the site of precipitation could lead to an increase in the boron isotopic composition of the marine carbonates even if the seawater pH was stable at this time. In contrast, we see a temporary decrease the B isotope composition across the T-OAE. Nevertheless, the transient temperature rise may have a minor effect on the B isotope composition, and minor temperature variations could be one of the factors causing the slight offset in $\delta^{11}B$ values between the two different basins.

<u>Salinity</u>: Similar to temperature, changes in salinity also affect the pK_B value[30]. To produce a pronounced negative shift in $\delta^{11}B$ values of marine carbonates, a drastic decrease in salinity is necessary (e.g., from 35 psu to 25 psu). However, models reconstructing the sea surface salinity changes during the T-OAE show that the areas around Spain and Portugal were not affected by salinity fluctuations[31]. In addition, both of our study areas represent shallow water environments away from direct freshwater inputs (see references above). Thus, an influence of salinity on the boron isotope composition of the marine carbonates is unlikely.

<u>Bathymetry</u>: As the depth in the water column increases, the temperature and hence the $\delta^{11}B$ values of the precipitated carbonate would decrease accordingly. Modern temperature profiles from the tropics show for example a drop of 15 °C in the first 300 m. In addition to temperature, the pH can decrease by up to ~0.2 units[32]. A potential depth effect on the B isotope composition is considered minor for both sections, due to the relatively stable depositional environment that is thought to have remained below storm-wave base throughout the studied time interva[16]. On the other hand, the greater paleo-depth at Portugal of about 200 m (6, 16) and likely colder (by

approximately 2 °C) seafloor water conditions[25] compared to Spain with a depth of 40 to 70 m, cannot account for the offset in the general $\delta^{11}B$ signature between both sites.

Weathering: While both sections are located around the Iberian Massif, they are situated on opposite margins (Fig. 1), which could lead to regional differences, not only in bathymetries, sedimentation rates, and water temperatures (see above), but also in weathering intensities. Enhanced weathering discharge from the continents during the T-OAE with varying magnitudes has already been suggested. For the eastern margin of the Panthalassa Ocean[33] and the northern parts of the NW Tethys[34], a threefold increase in weathering rates is proposed on the basis of e.g., osmium isotopes. Whereas for the Peniche area of the Lusitanian Basin in Portugal, five times stronger fluxes are reconstructed on the basis of calcium isotope compositions[35], leading to the turbiditic sedimentation observed here, which differs from that observed in the Rabaçal/Fonte Coberta section in the same Basin[10, 36]. The intensified continental weathering could have driven an increased ocean alkalinity and buffering capacity of the water in the potentially restricted basin[37, 38]. Corresponding weathering rates are, however, unknown for the Iberian Basin in Spain. An increased boron influx caused by enhanced continental weathering could have also led to a temporary decrease in the B isotope composition of seawater and thus to a decrease in the B isotopic composition in carbonates. However, given the residence time of the boron and the timing of the excursion, this is unlikely even in a potentially confined basin and would inevitably have had an impact on the B isotope composition of brachiopods and oysters.

Carbon dioxide: Rapid inputs of CO_2 and CH_4 into the atmosphere derived from LIP volcanism and the dissociation of marine clathrates at the onset of the T-OAE[39-43] have already been suggested to be a cause for a possible seawater acidification[44, 45, 46]. Climate models[47] estimated a rise in atmospheric CO_2 concentrations of about 2 to 4 times higher than pre-industrial levels. Such a rise in atmospheric CO_2 is supported by a study[25], using a Geocarb-style inversion model based on $\delta^{18}O$ values from brachiopod and bivalve shell material.

Source Material: In the Pliensbachian and Toarcian, skeletal material from macroinvertebrates derived most likely from brachiopods, bivalves, gastropods and ammonites. However, if we look at the boron mass fraction of the micrites, for example, these skeletal producers with their considerably higher boron mass fraction seem not responsible for most of the micritic carbonate production. There is also a clear, albeit small, difference in boron isotope composition. Comparatively low boron mass fractions are found in the opportunistic brachiopod species *Soaresirhynchia bouchardi*, which dominates the T-OAE. However, in contrast to the contemporaneous micrites, this species shows a substantially higher and constant boron isotope composition. In terms of sediment production, calcareous nannoplankton such as *Schizosphaerella*, coccoliths[48-52] and dinoflagellates[53-55] have a good record and may have produced most of the sampled micrite material. Planktonic foraminifera had yet to evolve and benthic foraminifera have a wide range in B mass fraction that can be close to the micrite but usually with higher contents[56, 57] of up to 25 $\mu g\ g^{-1}$ or even 65 $\mu g\ g^{-1}$. As such, a temporary contribution of benthic foraminifera should also be obvious via a change in the B mass fraction.

It is not known whether Schizosphaerella or dinoflagellates are able to record the pH value of seawater with or without being considerably influenced by vital effects. However, studies on the

B isotope composition of cultured and wild-grown coralline algae found a significant relationship between the $\delta^{11}B$ values and the pH of the seawater, but also observed an up-regulation of the calcifying fluid pH[58, 59] that is potentially species-specific[60]. A study on the B-isotope composition of coccolithophores[61] revealed different species-specific behaviours in the regulation of the pH of the calcifying fluid, where in addition to homeostasis, a relationship between changes in seawater pH and $\delta^{11}B$ values in the coccolith calcite was observed. In addition to Paris et al.[62], we also successfully analyzed microbial and abiotic micrites in the Neoproterozoic[28, 63] and the Permian Triassic Boundary[29], and found a significant relationship between the $\delta^{11}B$ values and the pH of the seawater that in case of the Permian Triassic Boundary is considered to record seawater $\delta^{11}B$ values in the absence of vital effects. While relationships between the $\delta^{11}B$ value of calcite and seawater pH have been observed for some of the assumed source materials in the micrite, there is no useful $\delta^{11}B$ ocean pH calibration for this mixture of sample types, and we can therefore only track trends in ocean pH conditions

While micritic material seems far from ideal to serve as a reliable $\delta^{11}B$-ocean pH archive, reproducing identical patterns in two sections of the same period but from different locations argues for an original $\delta^{11}B$ record. In addition, ancient micrites have been demonstrated to be a reliable archive for B isotope data to track trends in ocean pH conditions[28, 29, 62, 63], even if an accurate $\delta^{11}B$-ocean pH calibration and hence ocean pH assessment is not possible. Despite these uncertainties and problems, we calculated pH conditions from the B isotope composition of the micritic material (Fig. 3 and Supplementary Figure 4 to 9) using the relationship between pH and $\delta^{11}B_{borate}$ as detailed in the Model description in the Supplementary Methods (pH estimates from boron isotopes, equation 12).

Supplementary Methods

Model equations

Boundary conditions and flux formulations correspond, unless otherwise stated, to COPSE-reloaded "lowC_{in}" scenario, described in full in the original model (table 2, page 6[64]), incorporating minor modifications described by Tostevin and Mills[65]. This description will be restricted to changes from this original model formulation.

COPSE reloaded's default formulation for the atmospheric fraction $\phi_{(t)}$ of the global ocean atmosphere CO_2 reservoir A:

$$\phi_{(t)} = \phi_0 \cdot \frac{A_{(t)}}{A_0} \quad (1)$$

where $\phi_0 = 0.01614$ is the modern day atmospheric fraction, which was used to estimate surface ocean dissolved inorganic carbon concentration:

$$DIC_{(t)} = DIC_0 \cdot \frac{A_{(t)}}{A_0} \cdot (1 - \phi_{(t)}) \quad (2)$$

The sum of carbonate alkalinity and borate alkalinity was used to estimate total surface ocean alkalinity:

$$[TAlk]_{(t)} = TAlk_0 \cdot \left(\frac{CAlk_0}{TAlk_0} \cdot \frac{A_{(t)}}{A_0} \cdot \left(1 - \phi_{(t)}\right) + \frac{B_{(t)}}{B_0} \cdot \frac{BAlk_0}{TAlk_0}\right) \quad (3)$$

Where $CAlk_0 = [HCO_3^-]_0 + 2[CO_3^{2-}]_0$, with $[HCO_3^-]_0 = 1812\ \mu molL^{-1}$, $[CO_3^{2-}]_0 = 202\ \mu molL^{-1}$, $[BAlk]_0 = 92\ \mu molL^{-1}$, $TAlk_0 = 2400\ \mu molL^{-1}$. The model global average surface temperature T_{GAST} (COPSE reloaded, equation (2)) was assumed, following[29] to be 6.5°C lower than the sea surface temperature $T_{sea\ surface}$:

$$T_{sea\ surface(t)} = T_{GAST(t)} + 6.5 \quad (4)$$

Surface ocean phosphate concentration was approximated as scaling linearly with the global marine phosphate reservoir:

$$PO_4 = PO_{40} \cdot \frac{P}{P_0} \quad (5)$$

Where $PO_{40} = 2.2\ \mu molL^{-1}$. Finally, (and as per the original model, equation (1), reference (64)) the atmospheric CO_2 partial pressure varied with the square of the normalized global reservoir size $pCO_{2\ (atm)} = pCO_{2\ (atm)0} \cdot (\frac{A_{(t)}}{A_0})^2$, with pre-industrial value $pCO_{2\ (atm)0} = 280 ppm$. These simplifications (assuming modern surface pressure and salinity conditions) provided sufficient information for CO2SYS to calculate surface ocean pH, the inputs for which were $pCO_{2\ (atm)}$, $[TAlk]_{(t)}$, $T_{sea\ surface(t)}$, giving an estimate for surface ocean pH.

The pH estimate from CO2SYS was fed into the boron speciation function (equations (5.1) to (5.13), pages 108-110[56]), which was used to calculate K_B, pK_B and $x_B = \frac{[BOH_4^-]}{[BOH_4^-]+[BOH_3\ ]}$. The model prediction for surface/shelf water carbonate $\delta^{11}B$ composition was then calculated as:

$$\delta^{11}B_{carb} = \frac{\delta^{11}B_{seawater} - 1000(1 - x_B)(\alpha_B - 1)}{\alpha_B - x_B(\alpha_B - 1)} \quad (6)$$

With $\alpha_B = 1.0272$ (see "pH estimates from boron isotopes").

The mass of the global ocean-atmosphere CO_2 reservoir changed over time according to:

$$\frac{dA}{dt} = ocdeg + ccdeg + oxidw + carbw - mccb - mocb - tocb - sfw + F_{LIP\ CO_2} + F_{Clathrate\ CH_4} \quad (7)$$

Where the fluxes shown are, respectively, organic carbon degassing, carbonate carbon degassing, oxidative weathering, carbonate weathering, marine carbonate carbon burial, marine organic carbon burial, terrestrial organic carbon burial, seafloor "reverse" weathering, large igneous province associated CO_2 degassing input, and clathrate CH_4 degassing input (Supplementary Table 6). The isotopic composition δ_A of the atmosphere-ocean CO_2 pool changed according to:

$$\frac{d(\delta_A A)}{dt} = \delta_G(ocdeg + oxidw) + \delta_C(ccdeg + carbw) - \delta_A mccb - (\delta_A - \varepsilon)\cdot(mocb + tocb)$$

$$+\delta^{13}C_{CH_4}F_{Clathrate\ CH_4} + \delta^{13}C_{LIP}F_{LIP\ CO_2} \qquad (8)$$

Where δ_G and δ_C are the isotopic compositions of the organic and carbonate carbon reservoirs respectively. Marine carbonate carbon burial $mccb$ was assumed to balance silicate and carbonate weathering fluxes, thereby assuming steady state carbonate carbon:

$$mccb = silw + carbw \qquad (9)$$

Dynamics of the marine boron reservoir were described by the[66], equation (2):

$$\frac{dB}{dt} = B_{weath} + B_{IN} - B_{clastic} - B_{carbonate} - B_{crustlowTweath} \qquad (10)$$

We lump the boron influx from hydrothermal vents and fluid expulsion from accretionary prisms into a single influx term $B_{IN,0} = B_{accretion,0} + B_{hydro,0}$ (Table 6), which we tune for present day steady state reservoir size $\frac{dB}{dt} = 0$. The model seawater $\delta^{11}B$ isotopic composition changed via:

$$\frac{d(\delta^{11}B_{seawater}\cdot B)}{dt} = \delta^{11}B_{weath}\cdot B_{weath} + \delta^{11}B_{IN}\cdot B_{IN}$$

$$-(\delta^{11}B_{seawater} - \delta^{11}B_{clastic})\cdot B_{clastic} \ -(\delta^{11}B_{seawater} - \delta^{11}B_{carb})\cdot B_{carbonate}$$

$$-(\delta^{11}B_{seawater} - \delta^{11}B_{crustlowTweath})B_{crustlowTweath} \qquad (11)$$

Where the flux specific isotopic fractionations are selected from the measured range given by Lemarchand et al. (table 1[66]), with baseline modern value $\delta^{11}B_{seawater,0} = 39.61‰$, $\delta^{11}B_{weath} = 10‰$, $\delta^{11}B_{clastic} = 15‰$, $\delta^{11}B_{carb} = 20‰$, and $\delta^{11}B_{crustlowTweath} = 4‰$. The isotopic composition of the influx term was tuned for steady state present day mass and isotopic composition of the reservoir.

<u>Seawater pH estimates from boron isotopes</u>

Measured $\delta^{11}B$ data were fed into existing empirical inversion formulae, in order to provide an independent estimate for pH. The formula used from Klochko et al. (equation (18) [67]) was:

$$pH_{Klochko} = pK_B - log_{10}\left(\frac{\delta^{11}B_{seawater} - \delta^{11}B_{borate}}{\delta^{11}B_{seawater,0} - {}^{11-10}K_B\cdot\delta^{11}B_{borate} - 1000\cdot({}^{11-10}K_B - 1)}\right) \qquad (12)$$

where ${}^{11-10}K_B = 1.0272$, and we equate $\delta^{11}B_{borate} = \delta^{11}B_{micritic,data}$.

The inversion used by Jurikova et al. ([68] equation 5) was:

$$pH_{Jurikova} = pK_B - log_{10}\left(-\left(\frac{\delta^{11}B_{seawater} - \delta^{11}B_{borate}}{\delta^{11}B_{seawater} - {}^{11-10}K_B\cdot\delta^{11}B_{borate} - 1000\cdot({}^{11-10}K_B - 1)}\right)\right) \qquad (13)$$

The original work (equation (6)[67]) estimates $\delta^{11}B_{borate}$ in (13) as a function of the isotopic composition of brachiopod calcite $\delta^{11}B_{borate,Jurikova} = \frac{\delta^{11}B_{data}-11.52}{0.292}$. In order to avoid an unacceptable scatter when applied to our ancient, i.e., non-modern, seawater data, we introduced a slight modification to this fit:

$$\delta^{11}B_{borate,Jurikova} = \frac{\delta^{11}B_{data}-11.52+\delta^{11}B_{seawater,0}-\delta^{11}B_{seawater}}{0.292} \tag{14}$$

The formula from Lécuyer et al. ([69]) was applied with the assumption of fixed $pK_B = 8.9$:

$$pH_{Lécuyer} = 8.9 - log_{10}\left(\frac{0.023}{\frac{\delta^{11}B_{data}+1000}{\delta^{11}B_{seawater}+1000}-0.976} - 1\right) \tag{15}$$

The formula of Pennman et al.[70] (section 3.4) was

$$pH_{Penman} = pK_B - log_{10}\left(-\left(\frac{\delta^{11}B_{seawater}-\delta^{11}B_{data}-a}{\delta^{11}B_{seawater}-{}^{11-10}K_B\cdot(\delta^{11}B_{data}+a)-\varepsilon}\right)\right) \tag{16}$$

With $a = 2.4$, $\varepsilon = 27.2$.

<u>Temperature estimates from oxygen isotopes</u>

Four of the six temperature-$\delta^{18}O$ fits were of the form:

$$T_{\delta^{18}O\ (j)} = A - B\cdot\delta^{18}O_{data} + C\cdot({\delta^{18}O_{data}}^2) \tag{17}$$

With the values of the constants differing slightly according to the source (see Supplementary Table 7). The fit of Hansen et al.[71] specifically relates to benthic seawater of the sort inhabited by the brachiopods in this study:

$$\left(T_{\delta^{18}O\ (j)}\right)_{\delta^{18}O_{data}\geq 1.75} = -2\cdot(\delta^{18}O_{data} - 4.25),$$

$$(T_{\delta^{18}O})_{\delta^{18}O_{data}<1.75} = 12 - 4\cdot\delta^{18}O_{data} \tag{18}$$

The fit of Brand et al.[72] explicitly relates temperature to the difference between the $\delta^{18}O$ of shell calcite and seawater, and the impact on $\delta^{18}O$ of the proportion of $MgCO_3$ in the shell:

$$T_{\delta^{18}O\ (j)} = 16.192 - 3.48\cdot(\delta^{18}O_{data} - \delta^{18}O_{SW} - \delta_{Mg}) \tag{19}$$

Where the seawater average isotopic composition was assumed related to salinity Sal (provisionally fixed at $Sal = 35\ psu$, via the relationship derived by Marshall et al.[73]), giving $\delta^{18}O_{SW} = 0.417Sal - 15.118 = -0.593‰$. The magnesium calcite adjustment factor $\delta_{Mg} = 0.17\cdot mol\%MgCO_3$[72], which for this dataset was approximately $mol\%MgCO_3 = 0.4$.

Full model code is available at: https://github.com/richboyle111/COPSEBoron

		Spain (Submediterranean Province)		Portugal (Mediterranean Province)		
**Age of base**	**Stage**	**Zone**	**Subzone**	**Zone**	**Subzone**	**Age of base**
	middle Toarcian	bifrons	bifrons	bifrons	bifrons	
**180.39**			sublevisoni		sublevisoni	**180.39**
	early Toarcian	serpentinum	falciferum	levisoni	falciferum	
**182.92**			elegantulum		levisoni	**182.92**
		tenuicostatum	semicelatum II	polymorphum	semicelatum II	
**183.72**			paltum		mirabile	**183.72**
	late Pliensbachian	emaciatum	elisa	spinatum	hawskerense	
**184.55**			solare		apyrenum	
		algovianum	levidorsatum			
			meneghenii			**185.12**
			accuratum	margaritatus	gibbosus	
			bertrandi			
**186.15**			ragazzonii			
		lavinianum	cornacaldense		subnodosus	
**188.29**					stokesi	**188.29**

Supplementary Figure 1: Ammonite biostratigraphic zones. Ammonite biostratigraphic zones for Submediterranean (Spain) and Mediterranean provinces (Portugal)[74]. Ages given in million years (Ma) according to Geological Time Scale 2016[27].

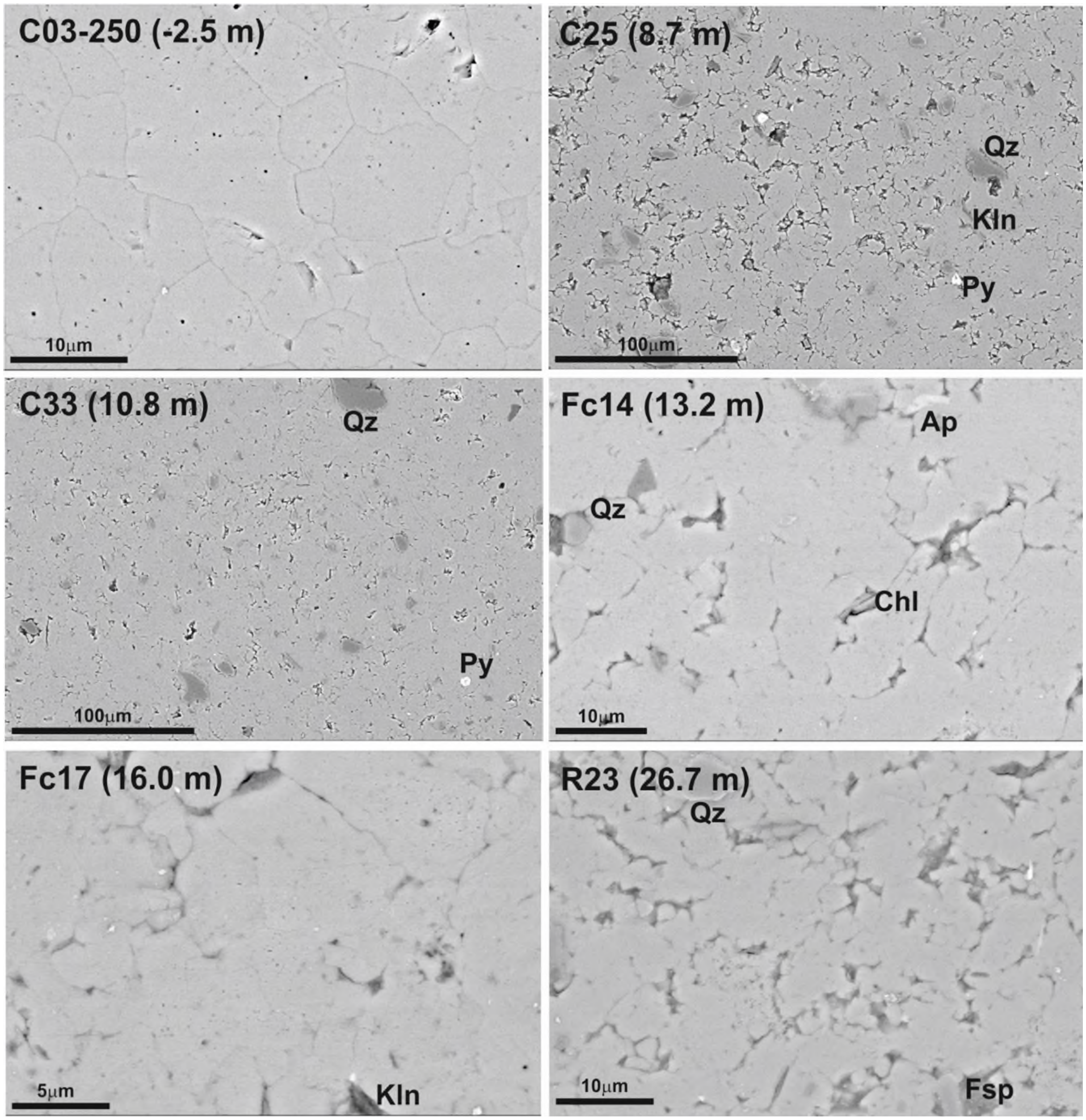

Supplementary Figure 2: Scanning electron microscope images of micrite. Examples of scanning electron microscope (SEM)/backscattered electrons (BSE) images for the Rabaçal/Fonte Coberta (Portugal) and for the Barranco de la Cañada (Spain) sections. Samples demonstrate homogeneous micritic texture of samples used for $\delta^{11}B$ analysis. Detrital components are quartz (Qz), feldspar, (Fsp), Kaolinite (Kln), Pyrite (Py) and chlorite (Chl). Stratigraphic interval and sample heights (m) given as in Supplementary Table 2 and 4.

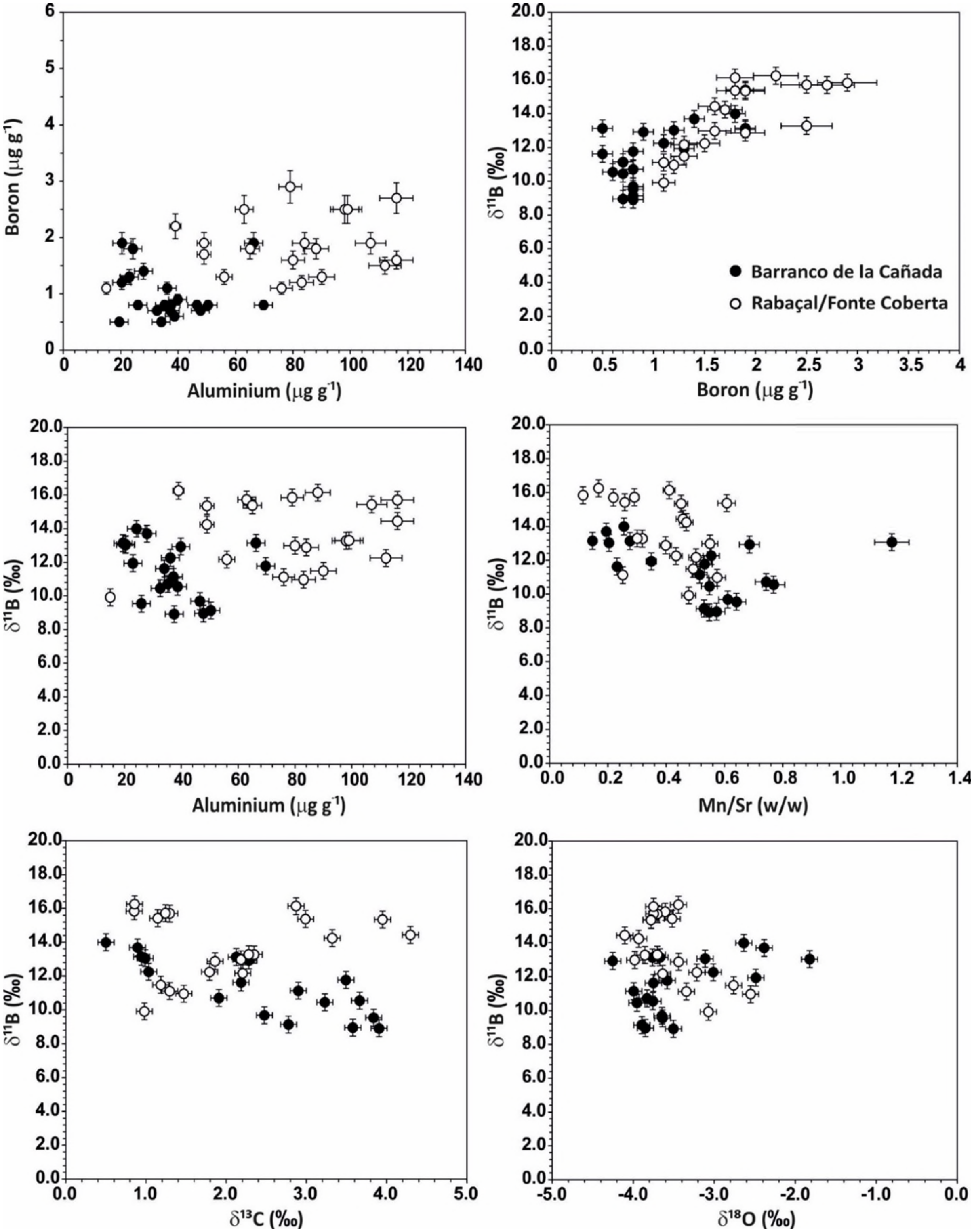

Supplementary Figure 3: Mass fraction and isotope cross-plots. Cross-plots of boron (2sd*f*), carbon, and oxygen isotope data (2sd), boron and aluminum mass fraction (2rsd), and Mn/Sr ratios for the marine micrite samples from the Rabaçal/Fonte Coberta (Portugal) and Barranco de la Cañada (Spain) sections.

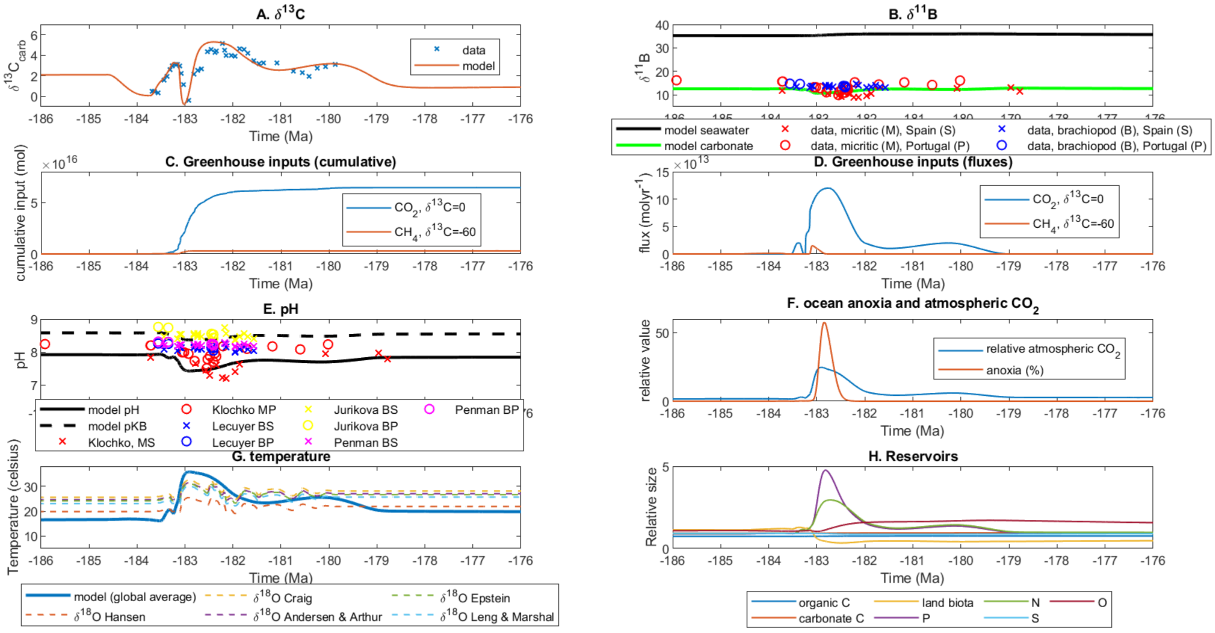

Supplementary Figure 4: Model output $\delta^{13}C$ = 0 ‰ for CO_2 and $\delta^{13}C$ = -60 ‰ for CH_4. Panel A compares the measured $\delta^{13}C$ data (blue diamond) with the model's estimate for surface ocean carbonate (solid red line). Panel B compares the measured $\delta^{11}B$ values (micritic carbonates and brachiopods) with model estimates of the seawater average (solid black line) and that associated with marine carbonate (solid green line). Panel C gives the cumulative greenhouse inputs (CO_2 solid blue line, CH_4 solid red line). Panel D gives the greenhouse fluxes (CO_2 solid blue line, CH_4 solid red line). Panel E compares the pH estimates derived from direct proxy inversion (colored symbols) with the model estimate for surface seawater pH (solid black line). Red symbols show micritic pH data (using the Klochko et al.[67] formula), other symbols and shades show brachiopod data (blue symbols[69], purple symbols[70], yellow symbols[68]). Proxy inversion estimates are a function of the model pK_B estimate (dashed black line), with the exception of that of[69], for which it was held constant at pK_B = 8.9. Panel F gives the relative values for CO_2 (solid blue line) and anoxia (%, solid red line). Panel G compares the temperature estimates from the model (solid blue line) to those derived from direct inversion of $\delta^{18}O$ values (colored dotted lines, the values of the constants and the sources are provided in Supplementary Table 7). Panel H gives the relative reservoirs sizes for organic carbon (solid blue line), carbonate carbon (solid orange line), nitrogen (solid green line), oxygen (solid red line), phosphorous (solid purple line), sulfur (solid light blue line) and land biota (solid yellow line). The combined input of isotopically neutral CO_2 and isotopically negative CH_4 was determined to be the best way to reproduce the $\delta^{13}C$ excursion and used for the model-data comparison in the main text.

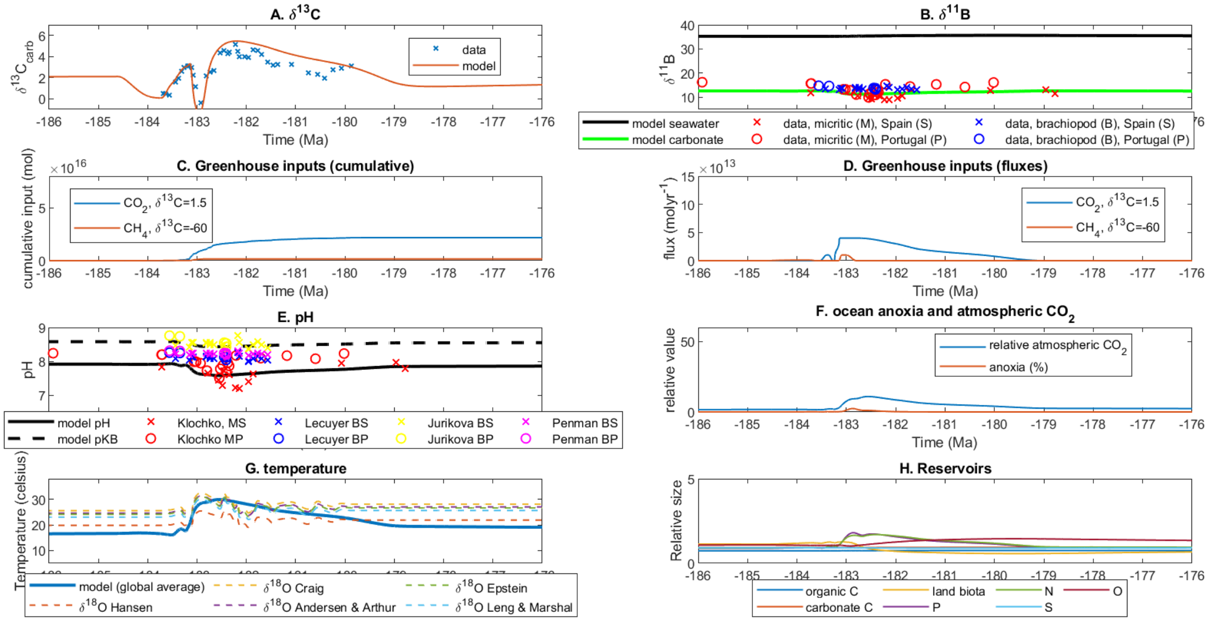

Supplementary Figure 5: Model output $\delta^{13}C$ = 1.5 ‰ for CO_2 and $\delta^{13}C$ = -60 ‰ for CH_4. Model outputs for isotopic composition inputs of $\delta^{13}C_{LIP}$ = 1.5 ‰ for large igneous province CO_2 and $\delta^{13}C_{CH4}$ = -60 ‰ for clathrate methane. Panels and the symbols are identical to Supplementary Figure 4.

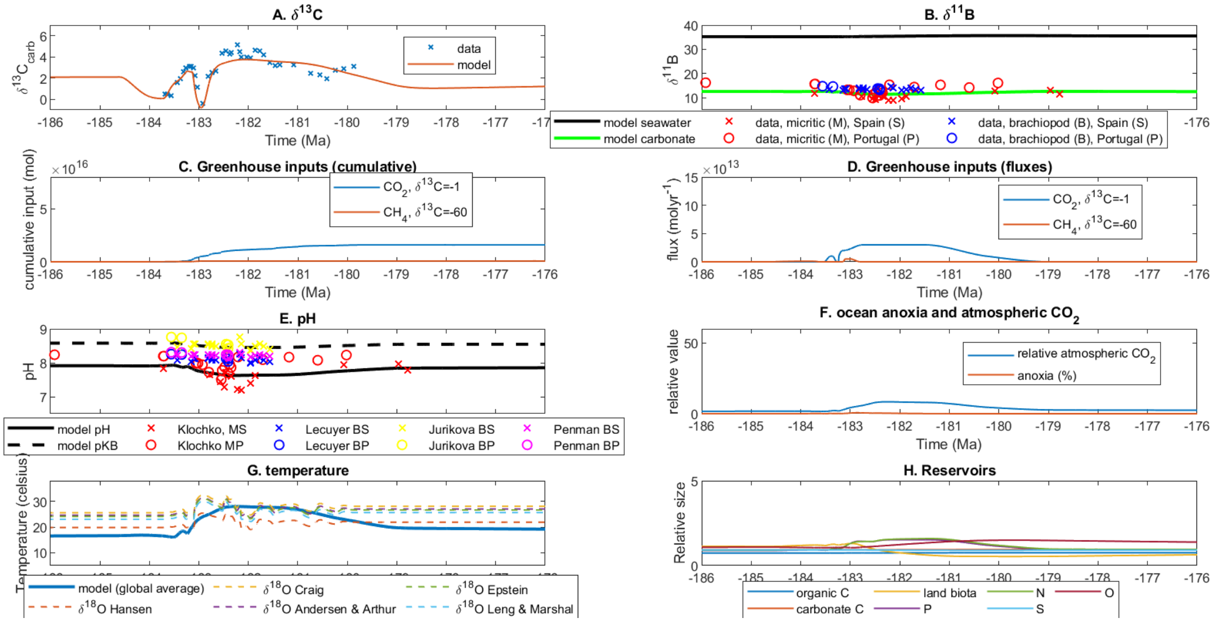

Supplementary Figure 6: Model output $\delta^{13}C$ = -1.0 ‰ for CO_2 and $\delta^{13}C$ = -60 ‰ for CH_4. Model outputs for isotopic composition inputs of $\delta^{13}C_{LIP}$ = -1.0 ‰ for large igneous province CO_2 and $\delta^{13}C_{CH4}$ = -60 ‰ for clathrate methane. Panels and the symbols are identical to Supplementary Figure 4.

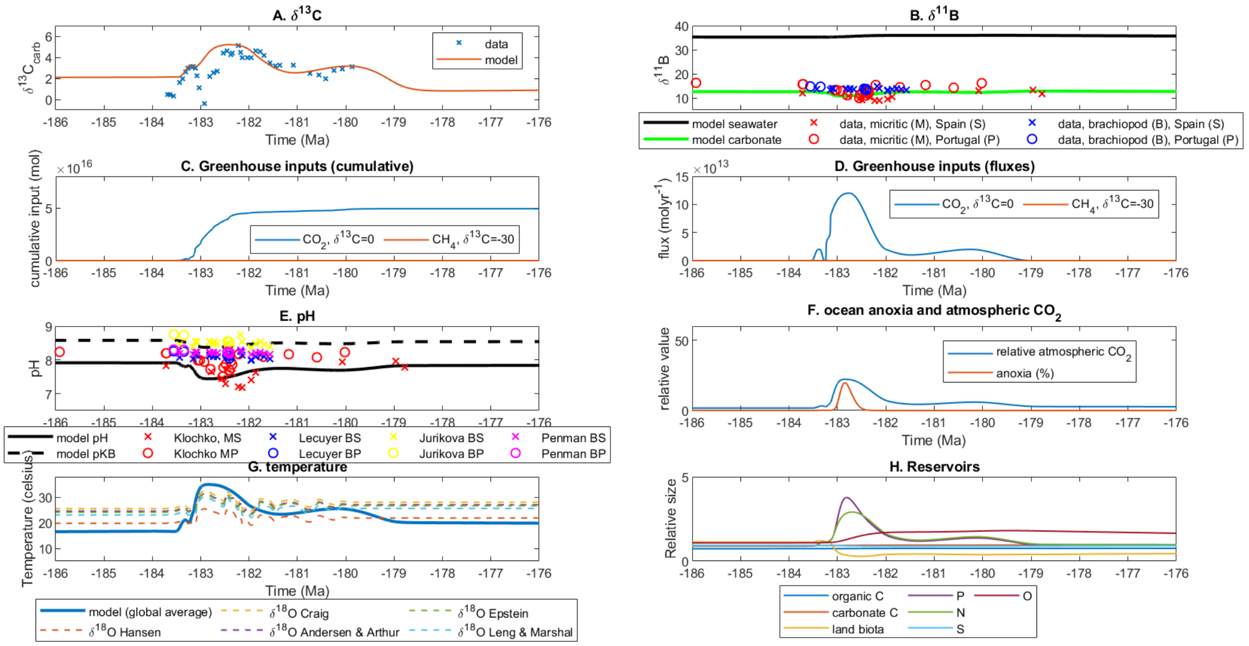

Supplementary Figure 7: Model output $\delta^{13}C$ = 0 ‰ for CO_2.

Model outputs for isotopic composition inputs of $\delta^{13}C_{LIP}$ = 0 ‰ for large igneous province CO_2 only, i.e., a model run without an isotopically negative greenhouse input. Panels and the symbols are identical to Supplementary Figure 4.

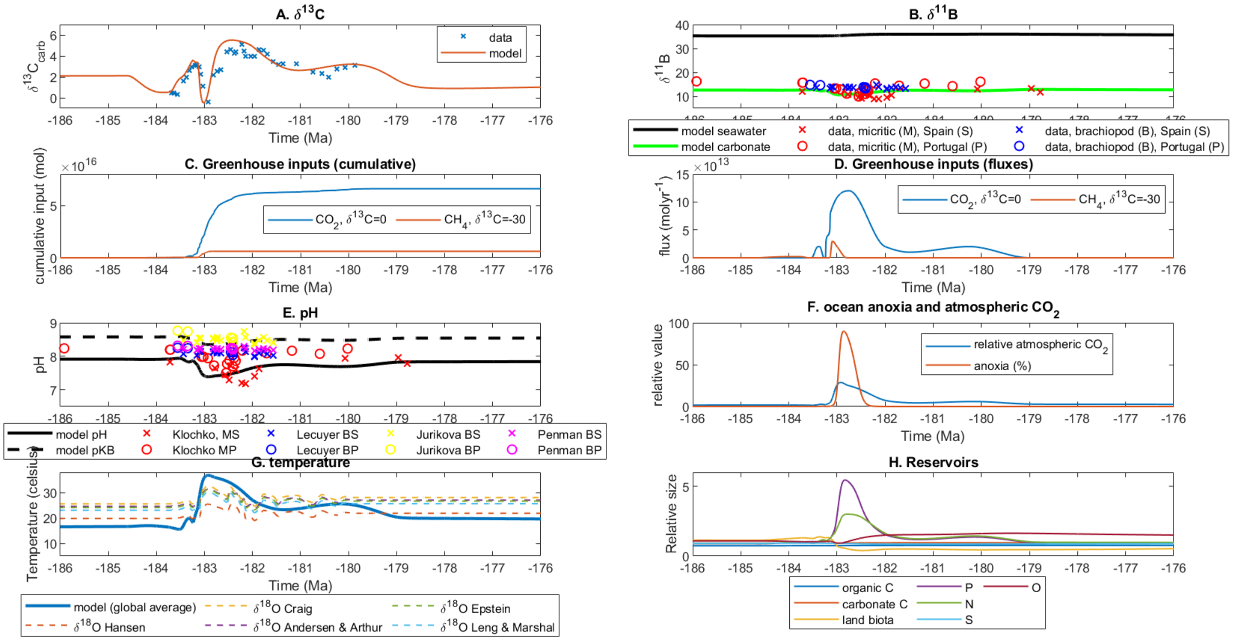

Supplementary Figure 8: Model output $\delta^{13}C$ = 0 ‰ for CO_2 and $\delta^{13}C$ = -30 ‰ for CH_4. Model outputs for isotopic composition inputs of $\delta^{13}C_{LIP}$ = 0 ‰ for large igneous province CO_2 and $\delta^{13}C_{CH4}$ = -30 ‰, corresponding to mixed thermogenic/volcanic sources. Panels and the symbols are identical to Supplementary Figure 4.

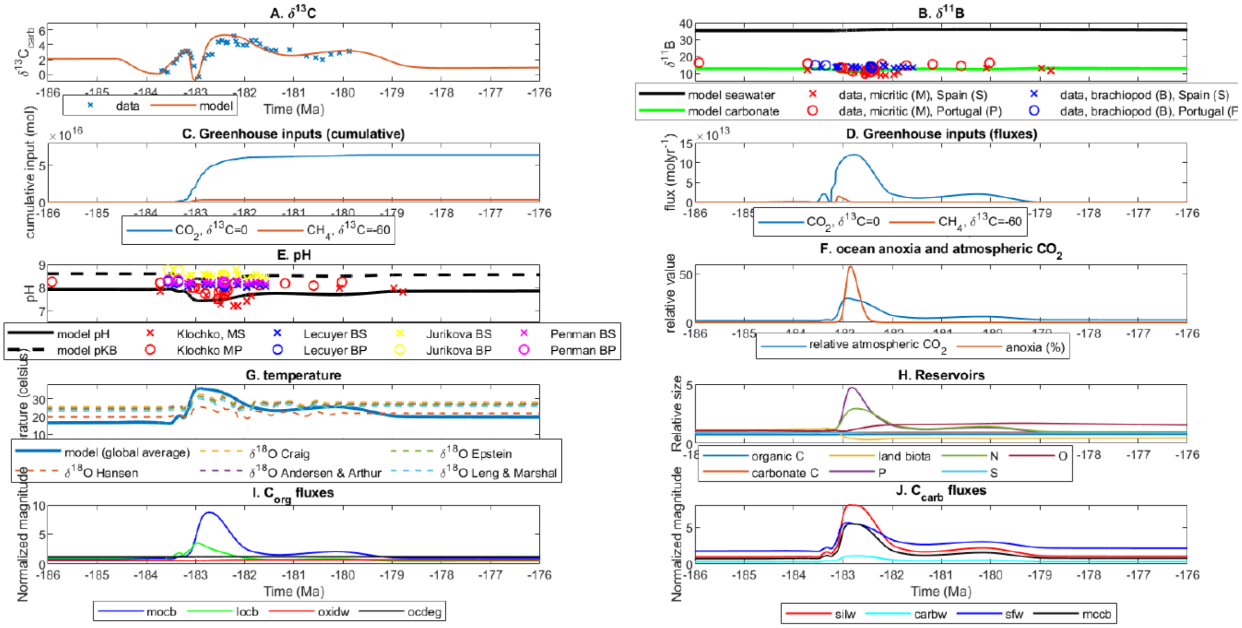

Supplementary Figure 9: Model output $\delta^{13}C$ = -1.0 ‰ for CO_2 and $\delta^{13}C$ = -60 ‰ for CH_4. Model outputs for isotopic composition inputs of $\delta^{13}C_{LIP}$ = -1.0 ‰ for large igneous province CO_2 and $\delta^{13}C_{CH4}$ = -60 ‰ for clathrate methane. Panels and symbols are identical to Supplementary Figure 4, but with two additional panels in the bottom row corresponding to the fluxes in the organic and carbonate-carbon derivatives, each normalized to their present value.

Sample code	Fossil type	Species	Height m	Age (Ma) GTS2016	$\delta^{13}C$ ‰	$\delta^{18}O$ ‰	$\delta^{11}B$ ‰	B $\mu g\ g^{-1}$	Ca wt%	Mg/Ca $mmol\ mol^{-1}$	Sr/Ca $mmol\ mol^{-1}$	Mn/Ca $mmol\ mol^{-1}$	Fe/Ca $mmol\ mol^{-1}$	S/Ca $mmol\ mol^{-1}$	P/Ca $mmol\ mol^{-1}$	$CaCO_3$ %
**Rabaçal/Fonte Coberta**																
FC10_4_1	B	*Cirpa fallax*	1.5	183.56	3.1	-1.3	14.8(1)	3	39	2.3	0.5	0.02	0.1	1.4	0.22	98
FC13a_1_1	B	*Cirpa fallax*	3.3	183.35	4.1	-1.3	14.7(1)	2	39	1.7	0.5	0.02	0.1	1.2	0.12	97
FC16e_1_1	B	*Soaresirhynchia bouchardi*	14.8	182.45	3.4	-2.5	13.8 (1)	1	39	3.0	0.5	0.02	0.2	1.7	0.12	100
FC17a_1_1	B	*Soaresirhynchia* cf. *flamandi*	15.4	182.41	3.1	-2.3	13.8(1)	1	39	2.4	0.5	0.01	0.1	1.5	0.18	99
FC17a_2_1	B	*Soaresirhynchia bouchardi*	15.4	182.41	3.1	-2.2	13.3(1)	1	39	3.5	0.5	0.02	0.2	1.6	0.18	100
**Barranco de la Cañada**																
C05_2_1	Biv	*Gryphaea (B.) sublobata*	0.4	183.69	1.2	-1.6	12.1(1)	3	39	1.8	0.6	0.03	0.4	7.3	0.21	99
C12a_3_1	B	*Quadratirhynchia attenuata*	2.8	183.43	1.5	-2.2	13.5(1)	51	38	5.3	1.0	0.02	0.2	4.7	0.03	99
C12a_2_1b	Biv	*Gryphaea (B.) sublobata*	2.8	183.43	2.1	-2.1	12.0(1)	12	39	2.8	0.8	0.03	0.5	4.8	0.00	99
C15a_1_1	Biv	*Gryphaea (B.) sublobata*	4.0	183.32	2.7	-2.2	12.2(1)	9	39	3.6	0.7	0.04	0.5	5.9	0.04	100
C18b_3_1	B	*Quadratirhynchia attenuata*	5.6	183.15	3.4	-1.6	13.3(1)	53	38	2.5	1.0	0.01	0.1	3.5	0.06	100
C18b_2_3	Biv	*Gryphaea (B.) sublobata*	5.6	183.15	3.9	-1.9	12.2(1)	8	39	4.4	0.8	0.02	0.6	2.1	0.00	101
C18c_1_3	B	*Quadratirhynchia* aff. *attenuata*	5.9	183.11	2.8	-2.0	13.9(2)	34	38	4.6	1.0	0.02	0.1	4.9	0.01	99
C18c_2_2	Biv	*Gryphaea (B.) sublobata*	5.9	183.11	3.8	-1.6	12.4(1)	9	39	2.9	0.8	0.01	0.3	1.5	0.04	101
C18c_2_3	Biv	*Gryphaea (B.) sublobata*	5.9	183.11	3.9	-1.4	12.5(1)	6	39	2.2	0.8	0.01	0.2	1.0	0.04	101
C19_4_1	B	*Quadratirhynchia attenuata*	6.2	183.08	2.2	-2.6	13.9(2)	73	38	4.8	1.0	0.02	0.2	5.3	0.15	98
C19_3_1	Biv	*Gryphaea (B.) sublobata*	6.2	183.08	3.1	-2.4	12.6(1)	10	39	3.4	0.7	0.04	0.5	4.1	0.02	100
C19_3_2	Biv	*Gryphaea (B.) sublobata*	6.2	183.08	3.0	-2.5	12.4(1)	8	39	3.2	0.7	0.02	0.3	6.1	0.05	99
C20_1_1	Biv	*Gryphaea (B.) sublobata*	6.7	183.04	1.8	-3.0	13.0(1)	7	39	4.4	0.8	0.04	0.4	3.2	0.15	100
C22_1_2	Biv	*Gryphaea* cf. *dumortieri*	7.8	182.92	0.4	-3.3	12.5(1)	7	39	3.3	0.8	0.05	0.7	1.2	0.24	100
C25a_2_2b	B	*Soaresirhynchia bouchardi*	8.4	182.82	2.2	-3.1	13.8(2)	2	39	3.5	0.5	0.05	0.4	1.4	0.00	100
C25a_2_1	B	*Soaresirhynchia bouchardi*	8.4	182.82	2.1	-3.0	13.9(2)	2	39	2.5	0.5	0.02	0.1	1.3	0.02	100
C25a_1_2	B	*Soaresirhynchia bouchardi*	8.4	182.82	2.5	-3.2	13.8(2)	2	39	2.3	0.5	0.02	0.1	1.3	0.01	100
C26_1_2	B	*Soaresirhynchia bouchardi*	8.9	182.74	2.3	-3.4	13.9(1)	1	39	2.7	0.5	0.02	0.1	1.4	0.02	100
C26_1_1	B	*Soaresirhynchia bouchardi*	8.9	182.74	2.3	-3.4	13.8(1)	1	39	2.5	0.5	0.02	0.1	1.2	0.05	100
C26_1_3	B	*Soaresirhynchia bouchardi*	8.9	182.74	2.8	-2.5	13.8(2)	1	39	3.4	0.6	0.01	0.1	1.5	0.00	101
C26_1_5	B	*Soaresirhynchia bouchardi*	8.9	182.74	2.3	-3.2	13.6(2)	1	39	2.1	0.5	0.01	0.0	1.1	0.00	101
C28_1_3	B	*Soaresirhynchia bouchardi*	9.4	182.66	2.8	-2.6	13.6(2)	1	39	2.6	0.5	0.01	0.1	1.3	0.04	101
C33a_1_1	B	*Soaresirhynchia bouchardi*	10.8	182.45	4.6	-3.4	13.6(1)	2	39	2.8	0.5	0.02	0.3	1.4	0.02	101
BC01b_2_1	B	*Choffatirhynchia vasconcellosi*	12.4	182.18	4.5	-2.8	14.8(1)	21	38	5.2	0.9	0.08	0.4	2.9	0.11	100
BC02_3_1	B	*Choffatirhynchia* aff. *paucicostatae*	12.7	182.14	3.9	-2.1	13.9(1)	28	38	7.1	1.0	0.07	0.4	3.9	0.13	100
BC04_2_1	B	*Pseudogibbirhynchia* aff. *jurensis*	13.8	181.96	3.8	-1.8	13.0(1)	40	38	4.7	1.1	0.05	0.1	2.7	0.11	99
BC04_3_3	B	*Homoeorhynchia?* aff. *batalleri*	13.8	181.96	4.2	-1.4	13.0(1)	29	38	5.1	1.0	0.05	0.1	2.8	0.19	99
BC05_1_1	B	*Homoeorhynchia batalleri*	14.3	181.87	4.6	-2.0	13.6(1)	40	39	4.7	1.1	0.08	0.3	2.4	0.09	99
BC06_2_1	B	*Homoeorhynchia meridionalis*	14.9	181.77	4.6	-3.1	14.0(2)	27	38	4.7	1.0	0.04	0.2	3.1	0.18	99
BC06_1_1	Biv	*Gryphaea* cf. *dumortieri*	14.9	181.77	5.1	-2.5	12.1(1)	9	39	3.0	0.9	0.06	0.4	1.4	0.04	100
BC06_1_1	Biv	*Gryphaea* cf. *dumortieri*	14.9	181.77	5.3	-2.5	12.0(1)	9	39	3.1	0.9	0.05	0.4	0.9	0.05	100
BC07_1_2	B	*Homoeorhynchia meridionalis*	15.3	181.69	4.1	-2.6	13.6(1)	33	38	3.4	1.0	0.05	0.3	2.4	0.13	98
BC08_2_1	B	*Homoeorhynchia batalleri*	16.1	181.57	3.6	-2.3	13.2(2)	44	38	3.8	1.1	0.05	0.1	3.1	0.15	99
BC10_1_1	Biv	*Gryphaea (B.) sublobata*	17.3	181.37	3.9	-2.6	11.5(1)	11	39	2.4	0.8	0.06	0.4	1.2	0.12	101

Supplementary Table 1: Element mass fractions and isotope composition for fossil shells. Element mass fractions, element/Ca ratios and carbon, oxygen and boron isotope composition for bivalves (Biv) and brachiopods (B). The uncertainty (2sd) for the B isotope data is valid for the last digit. Sample code includes stratigraphic interval, sample ID and specimen number as provided by Ullmann et al.[25]. Heights refer to the Pl-To boundary. Boron isotope and mass fraction data are from this study; all other data and information are published[25].

Stratigraphic interval	Height m	Age (Ma) GTS2016	$\delta^{13}C$ ‰	$\delta^{18}O$ ‰	$\delta^{11}B$ ‰
**Rabaçal/Fonte Coberta**					
FC - Z10	-15.3	187.34	1.1	-3.5	15.4(4)
FC - Z23	-11.8	186.16	0.9	-3.6	15.8(1)
FC - Z29	-11.1	185.92	0.9	-3.4	16.2(1)
FC06	0.0	183.72	1.3	-3.7	15.7(1)
FC06	0.1	183.71	1.2	-3.7	15.7(1)
FC13	6.0	183.06	2.3	-3.9	13.3(1)
FC13	6.3	183.02	2.3	-3.7	13.3(2)
FC14	7.1	182.93	1.9	-3.4	12.9(1)
FC14	9.2	182.80	1.3	-3.3	11.1(2)
FC14	13.2	182.55	1.0	-3.1	9.9(2)
FC14	13.4	182.53	1.2	-2.8	11.5(1)
FC14	14.4	182.48	1.8	-3.2	12.2(1)
FC14	15.4	182.41	1.5	-2.6	11.0(1)
FC17	16.0	182.38	2.2	-4.0	13.0(1)
FC17	16.3	182.35	2.2	-3.6	12.2(1)
FC17	18.7	182.21	3.0	-3.8	15.4(1)
R23	26.7	181.71	4.3	-4.1	14.4(1)
R30	35.2	181.18	3.9	-3.8	15.3(1)
R30	44.6	180.60	3.3	-3.9	14.2(1)
R40	53.9	180.02	2.9	-3.7	16.1(1)
**Barranco de la Cañada**					
C03-1180	-11.8	188.02	0.9	-3.7	13.1(1)
C03-1080	-10.8	187.89	1.0	-3.0	12.3(1)
C03-540	-5.4	187.21	1.0	-3.1	13.1(1)
C03-354	-3.5	186.97	0.9	-2.4	13.7(1)
C03-250	-2.5	186.84	-0.1	-1.8	13.0(1)
C03-150	-1.5	186.71	0.5	-2.6	14.0(1)
C03	0.0	183.72	0.0	-2.5	11.9(1)
C25	8.7	182.78	1.9	-3.8	10.7(1)
C28	10.0	182.56	2.5	-3.6	9.7(1)
C31/C32	10.5	182.49	2.8	-3.9	9.1(1)
C33	10.7	182.45	2.9	-4.0	11.1(1)
C37	11.4	182.34	3.2	-4.0	10.5(1)
C37/BC01	11.8	182.28	3.5	-3.6	11.8(1)
BC01	12.1	182.23	3.6	-3.8	9.0(1)
BC02	12.6	182.14	3.9	-3.5	8.9(2)
BC04	13.7	181.96	3.8	-3.6	9.5(1)
BC5	14.3	181.87	3.7	-3.8	10.5(2)
BC21	25.3	180.08	2.3	-4.3	12.9(2)
BC26	32.2	178.96	2.1	-3.8	13.1(1)
BC27	33.4	178.78	2.2	-3.8	11.6(1)

Supplementary Table 2: Isotope compositions for micrites. Carbon, oxygen and boron isotope compositions for micrite samples from Rabaçal/Fonte Coberta (Portugal) and Barranco de la Cañada (Spain). The uncertainty (2sd) for the B isotope data is valid for the last digit (conservative rounding). The stratigraphic interval code is according to Ullmann et al.[25]. Heights refer to the Pl-To boundary.

Stratigraphic interval	Species	Ca wt%	Mg/Ca mmol mol^{-1}	Sr/Ca mmol mol^{-1}	Na/Ca mmol mol^{-1}	Mn/Ca mmol mol^{-1}	Fe/Ca mmol mol^{-1}	S/Ca mmol mol^{-1}	P/Ca mmol mol^{-1}	Al/Ca mmol mol^{-1}	Rb/Ca mmol mol^{-1}
**Rabaçal/Fonte Coberta**											
FC10	*Cirpa fallax*	39.0	2.28	0.535	1.98	0.022	0.08	1.39	0.27	0.14	d.l.
FC10	*Cirpa fallax*	38.8	2.34	0.524	1.75	0.016	0.04	1.28	0.29	0.10	d.l.
FC13a	*Cirpa fallax*	38.9	2.25	0.538	2.02	0.015	0.11	1.43	0.12	0.08	d.l.
R23c	*Homoeorhynchia meridionalis*	38.6	6.71	0.941	6.08	0.032	0.32	2.67	0.26	0.26	d.l.
R23c	*Homoeorhynchia meridionalis*	38.4	5.07	0.961	6.67	0.024	0.22	2.02	0.16	0.15	d.l.
R23c	*Homoeorhynchia meridionalis*	38.6	5.38	0.960	6.71	0.028	0.26	2.07	0.14	0.22	d.l.
FC13e	*Nannirhynchia pygmaea*	38.9	1.36	0.550	1.89	0.009	0.02	0.91	0.09	0.13	d.l.
FC14c	*Nannirhynchia pygmaea*	38.9	2.22	0.526	1.65	0.052	0.28	0.99	0.08	0.24	d.l.
FC14b	*Nannirhynchia pygmaea*	38.8	2.06	0.555	1.87	0.028	0.15	1.06	0.07	0.10	d.l.
FC16d	*Soaresirhynchia bouchardi*	38.6	3.45	0.502	1.21	0.048	1.03	1.18	0.14	0.20	d.l.
FC17a	*Soaresirhynchia bouchardi*	38.8	3.50	0.506	1.23	0.014	0.15	1.33	0.05	0.10	d.l.
FC17d	*Soaresirhynchia bouchardi*	39.2	2.05	0.509	1.09	0.009	0.17	0.84	0.15	0.10	d.l.
FC17a	*Soaresirhynchia* cf. *flamandi*	38.3	2.44	0.520	1.27	0.096	8.49	1.09	0.06	0.08	d.l.
FC17a	*Soaresirhynchia* cf. *flamandi*	39.1	2.60	0.506	1.12	0.008	0.01	1.10	0.02	0.07	d.l.
FC17a	*Soaresirhynchia* cf. *flamandi*	39.1	2.67	0.504	1.11	0.008	0.00	1.27	0.16	0.15	d.l.
**Barranco de la Cañada**											
BC02	*Choffatirhynchia* aff. *paucicostatae*	38.2	8.00	1.014	6.83	0.057	0.26	3.90	0.26	0.16	d.l.
BC02	*Choffatirhynchia* aff. *paucicostatae*	38.4	7.10	0.930	6.09	0.063	0.30	3.48	0.22	0.12	d.l.
BC02	*Choffatirhynchia* aff. *paucicostatae*	38.1	6.41	0.978	6.61	0.055	0.24	3.32	0.22	0.18	d.l.
BC02	*Choffatirhynchia vasconcellosi*	38.3	7.50	0.990	7.14	0.058	0.27	3.76	0.21	0.10	d.l.
BC02	*Choffatirhynchia vasconcellosi*	38.4	7.89	1.014	7.22	0.050	0.28	3.91	0.23	0.10	d.l.
BC02	*Choffatirhynchia vasconcellosi*	38.4	8.43	1.017	8.02	0.066	0.37	4.39	0.18	0.15	d.l.
C05	*Gibbirhynchia* cf. *cantabrica*	38.2	5.39	1.146	9.51	0.017	0.04	8.31	0.18	0.09	d.l.
C05	*Gibbirhynchia* cf. *cantabrica*	38.2	5.02	1.140	9.35	0.013	0.05	7.89	0.16	0.07	d.l.
C05	*Gibbirhynchia* cf. *cantabrica*	38.2	4.45	1.060	8.37	0.022	0.07	7.32	0.16	0.14	d.l.
BC04	*Gibbirhynchia muirwoodae*	38.1	5.40	1.132	8.51	0.054	0.13	2.95	0.15	0.10	d.l.
BC04	*Gibbirhynchia muirwoodae*	38.4	4.79	1.072	7.66	0.045	0.09	2.53	0.13	0.12	d.l.
BC04	*Gibbirhynchia muirwoodae*	38.5	5.62	1.136	8.61	0.064	0.20	2.73	0.23	0.13	d.l.
BC05	*Homoeorhynchia batalleri*	38.1	4.16	0.999	7.16	0.086	0.27	2.49	0.22	0.13	d.l.
BC03	*Homoeorhynchia batalleri*	38.9	6.25	0.911	5.87	0.089	0.41	3.51	0.05	0.11	d.l.
BC03	*Homoeorhynchia batalleri*	37.2	7.11	0.993	8.50	0.046	0.27	4.05	0.20	0.10	d.l.
BC07	*Homoeorhynchia meridionalis*	38.6	6.26	0.959	6.38	0.103	0.51	3.27	0.21	0.10	d.l.
BC07	*Homoeorhynchia meridionalis*	38.6	4.19	0.951	6.41	0.101	0.38	2.61	0.20	0.13	d.l.
BC07	*Homoeorhynchia meridionalis*	38.5	4.56	0.951	6.84	0.097	0.45	2.70	0.24	0.07	d.l.
C18c	*Quadratirhynchia* aff. *attenuata*	37.8	6.03	1.100	8.34	0.012	0.09	5.09	0.12	0.08	d.l.
C18c	*Quadratirhynchia* aff. *attenuata*	38.3	3.61	0.954	6.89	0.021	0.25	3.42	0.06	d.l.	d.l.
C18c	*Quadratirhynchia* aff. *attenuata*	37.4	5.49	1.070	8.13	0.009	0.09	5.01	0.15	0.10	d.l.
C18b	*Quadratirhynchia attenuata*	37.9	3.16	1.022	7.89	0.014	0.11	3.23	0.10	0.14	d.l.
C19	*Quadratirhynchia attenuata*	38.4	6.05	0.949	6.68	0.046	0.58	5.20	0.14	0.22	d.l.
C19	*Quadratirhynchia attenuata*	38.0	5.66	0.986	7.16	0.032	0.35	4.99	0.18	0.18	d.l.
C26	*Soaresirhynchia bouchardi*	38.9	2.34	0.531	1.18	0.009	0.04	1.29	0.09	0.14	d.l.
C26	*Soaresirhynchia bouchardi*	39.2	2.02	0.562	1.22	0.008	0.04	1.21	0.08	0.08	d.l.
C26	*Soaresirhynchia bouchardi*	38.9	2.34	0.543	1.22	0.011	0.07	1.25	0.12	0.06	d.l.
C33a	*Soaresirhynchia bouchardi*	39.1	3.11	0.551	1.24	0.007	0.03	1.31	0.08	0.09	d.l.
C25b	*Soaresirhynchia bouchardi*	34.5	2.36	0.522	1.21	0.026	0.16	1.17	0.12	0.09	d.l.
C37a	*Soaresirhynchia bouchardi*	39.0	3.52	0.519	1.36	0.036	0.31	1.36	0.15	0.08	d.l.

Supplementary Table 3: Element mass fraction for brachiopod shells. Additional element mass fraction and element/Ca ratios for brachiopods from Portugal and Spain. Below detection limit (d.l.). Stratigraphic interval as provided by Ullmann et al.[25].

Rabaçal/Fonte Coberta

Stratigraphic interval	Height m	Detritus wt%	Al $\mu g\ g^{-1}$	B $\mu g\ g^{-1}$	Ba $\mu g\ g^{-1}$	Ca wt%	Fe $\mu g\ g^{-1}$	K $\mu g\ g^{-1}$	Mg $\mu g\ g^{-1}$	Mn $\mu g\ g^{-1}$	Si $\mu g\ g^{-1}$	Sr $\mu g\ g^{-1}$	Mn /Sr w/w	Mg /Ca w/w
FC - Z10	-15.3	9	107	1.9	5.7	40	1070	547	3256	98	404	380	0.3	0.01
FC - Z23	-11.8	16	79	2.9	6.6	40	836	719	3915	73	348	638	0.1	0.01
FC - Z29	-11.1	13	39	2.2	10.9	40	803	617	3279	78	123	464	0.2	0.01
FC06	0.0	14	116	2.7	6.8	40	1467	694	3871	117	401	535	0.2	0.01
FC06	0.1	11	63	2.5	4.6	40	1274	521	3501	119	280	409	0.3	0.01
FC13	6.0	15	98	2.5	11.1	40	2223	711	3790	140	367	440	0.3	0.01
FC13	6.3	22	99	2.9	7.2	40	1792	846	3712	146	383	486	0.3	0.01
FC14	7.1	16	84	1.9	15	40	2253	655	3150	171	358	430	0.4	0.01
FC14	9.2	8	76	1.1	2	40	2923	481	3297	117	304	466	0.3	0.01
FC14	13.2	13	15	1.1	2.4	40	1667	592	3365	149	150	312	0.5	0.01
FC14	13.4	10	90	1.3	2.3	40	2189	544	3709	166	347	337	0.5	0.01
FC14	14.4	11	112	1.5	3.4	40	3167	605	3886	170	436	392	0.4	0.01
FC14	15.4	8	83	1.2	2.3	40	3269	535	3993	207	350	360	0.6	0.01
FC17	16.0	16	80	1.6	3.2	40	3109	741	3798	176	384	320	0.6	0.01
FC17	16.3	12	56	1.3	3.6	40	3376	585	4108	195	266	388	0.5	0.01
FC17	18.7	16	65	1.8	5.4	40	3001	849	3673	183	382	301	0.6	0.01
R23	26.7	13	116	1.6	3	40	2609	606	3683	145	379	317	0.5	0.01
R30	35.2	16	49	1.9	4	40	2452	761	4101	167	284	370	0.5	0.01
R30	44.6	15	49	1.7	4.7	40	2890	698	4360	176	246	375	0.5	0.01
R40	53.9	17	88	1.8	3.5	40	2070	801	3971	137	426	334	0.4	0.01

Barranco de la Cañada

Stratigraphic interval	Height m	Detritus wt%	Al $\mu g\ g^{-1}$	B $\mu g\ g^{-1}$	Ba $\mu g\ g^{-1}$	Ca wt%	Fe $\mu g\ g^{-1}$	K $\mu g\ g^{-1}$	Mg $\mu g\ g^{-1}$	Mn $\mu g\ g^{-1}$	Si $\mu g\ g^{-1}$	Sr $\mu g\ g^{-1}$	Mn /Sr w/w	Mg /Ca w/w
C03-1180	-11.8	4	66	1.9	1.5	40	851	125	3491	41	86	279	0.1	0.01
C03-1080	-10.8	5	36	1.1	1.6	40	543	171	3501	36	65	274	0.1	0.01
C03-540	-5.4	2	20	1.9	44.0	40	693	82	3686	47	40	251	0.2	0.01
C03-354	-3.5	4	28	1.4	1.4	40	488	176	4197	43	46	224	0.2	0.01
C03-250	-2.5	4	20	1.2	1.1	40	506	115	5003	44	31	217	0.2	0.01
C03-150	-1.5	3	24	1.8	1.6	40	600	133	4007	54	42	210	0.3	0.01
C03	0.0	6	23	1.3	6.3	40	1003	169	4995	118	36	338	0.3	0.01
C25	8.7	9	35	0.8	3.1	40	2057	242	4300	231	53	311	0.7	0.01
C28	10.1	12	47	0.8	9.9	40	2011	332	4274	215	54	353	0.6	0.01
C31/C32	10.5	14	50	0.8	9.1	40	2047	382	4392	209	55	394	0.5	0.01
C33	10.8	11	37	0.7	6.8	40	2170	319	4265	204	32	396	0.5	0.01
C37	11.4	14	33	0.7	7.7	40	2060	323	4291	211	88	385	0.5	0.01
C37/BC01	11.8	17	70	0.8	6.5	40	1531	333	4430	207	211	389	0.5	0.01
BC01	12.1	14	48	0.7	7.6	40	2105	373	4314	224	97	390	0.6	0.01
BC02	12.6	13	37	0.8	5.2	40	2094	381	4506	205	86	375	0.5	0.01
BC04	13.7	10	26	0.8	7.0	40	2123	357	4364	239	51	372	0.6	0.01
BC5	14.3	13	39	0.6	8.9	40	2692	386	4423	269	85	350	0.8	0.01
BC21	25.4	13	40	0.9	7.3	40	2245	365	4497	263	75	383	0.7	0.01
BC26	32.2	5	19	0.5	2.5	40	465	202	3609	118	42	428	0.3	0.01
BC27	33.4	5	34	0.5	2.3	40	374	164	3500	99	60	427	0.2	0.01

Supplementary Table 4: Element mass fraction and detrital content data for micrites. Carbonate samples are from Rabaçal/Fonte Coberta (Portugal) and Barranco de la Cañada (Spain). The stratigraphic interval code is according to Ullmann et al.[25]. Heights refer to the Pl-To boundary, i.e., specimens with negative meters are from the Pliensbachian.

Stratigraphic interval	Detrital wt%	Calcite wt%	Quartz wt%	Mica wt%	Chlorite wt%	Kln-Serp Group wt%	Feldspar Group wt%	Sulphides wt%
FC - Z10	9	81	9	5		4		1
FC06	14	69	13	5	3		6	3
FC13	22	74	17	12		2	7	
FC14	8	76	8	4		6	5	
FC14	13	83	9	4		4		
FC14	11	91	4	4		1		
FC17	12	87	8	3		2		
FC17	16	75	14	5		5		
R23	13	72	11	4		9	4	
R30	16	79	13	6		3		

Supplementary Table 5: XRD results of mineralogical composition. Results of some selected samples from Rabaçal/Fonte Coberta (Portugal). The weight percentage of detrital content was determined by carbonate leaching.

Abbreviation	Meaning	Modification from source model for this study
$carbw$	Carbonate weathering flux from land surface	n/a
$ccdeg$	CO_2 degassing flux from the subduction of carbonate	n/a
$ocdeg$	CO_2 degassing flux from the subduction of organic carbon in rock	n/a
$mccb$	Marine carbonate carbon burial flux	n/a
$mocb$	Marine organic carbon burial flux	n/a
$oxidw$	Oxidative weathering flux of organic carbon in rock on the land surface	n/a
$\delta^{13}C_{mccb}$	Isotopic composition of carbonates precipitating in shallow shelf ocean waters (assumed equilibrated with ocean-atmosphere CO_2 reservoir)	n/a
ε	Difference between the isotopic composition of the marine organic carbon burial flux $mocb$ and the marine carbonate carbon burial flux $mccb$. Expressed as a positive number $\varepsilon > 0$, such the isotopic composition of organic matter being buried in shallow ocean sediments is $\delta^{13}C_{org} = \delta^{13}C_{mccb} - \varepsilon < 0$	n/a
F_{LIP}	CO_2 release flux associated with large igneous province eruption. Parameterized.	Parameterized
$\delta^{13}C_{LIP}$	Isotopic composition of large igneous province derived CO_2. $\delta^{13}C_{LIP} = 0$	Parameterized, $(\delta^{13}C_{LIP})_{syn-volcanic} = -5‰$,[75] $-27 \leq (\delta^{13}C_{LIP})_{Thermogenic} \leq 2‰$[76]

F_{CH_4}	CO_2 release flux associated with the oxidation of CH_4 produced during clathrate decomposition. Parameterized	Parameterized, see text
$\delta^{13}C_{CH_4}$	Isotopic composition of CH_4-clathrate	$\delta^{13}C_{CH_4} = -60‰$, with the exception of Supplementary Figure 8 for which $\delta^{13}C_{CH_4} = -30‰$,
$ccdeg_0$	Baseline carbonate carbon degassing flux	n/a
A	Total ocean-atmosphere CO_2 reservoir size	n/a
ϕ	Atmospheric CO_2 fraction	n/a
B_{weath}	Boron weathering input	$B_{weath} = W \cdot B_{weath,0}$ where $B_{weath,0} = 3.5153 \times 10^{10} molyr^{-1}$[66].
$B_{IN} = B_{accretion} + B_{hydro}$	Combined input flux for steady state (fluid expelled from accretionary prisms plus hydrothermal input to oceans)	$B_{IN} = B_{IN0} \cdot D$ Steady state, i.e. $B_{IN0} = B_{clastic0} + B_{crustlowTweath0} + B_{carbonate0} - B_{weath,0}$ giving $B_{IN0} = 7.40 \times 10^9 molyr^{-1}$ (comparing adequately with[66]) $B_{accretion,0} = 1.8501 \times 10^9 molyr^{-1}$ and $B_{hydro,0} = 3.7 \times 10^9 molyr^{-1}$)
$B_{accretion}$	Boron input via fluid expelled from accretionary prisms.	$B_{accretion,0} = 1.8501 \times 10^9 molyr^{-1}$, $B_{accretion} = B_{accretion,0} \cdot D \cdot f_{runoff}$, where D is the COPSE degassing forcing, proportional to seafloor spreading rate, and f_{runoff} is the temperature weathering dependency from COPSE reloaded (equation 25[64])
B_{hydro}	Hydrothermal Boron input	$B_{hydro,0} = 3.7 \times 10^9 molyr^{-1}$, $B_{hydro} = B_{hydro,0} \cdot D$
$B_{clastic}$	Boron deposition via adsorption on to clastic materials	$B_{clastic,0} = 1.203 \times 10^{10} molyr^{-1}$ $B_{clastic} = B_{clastic,0} \cdot \frac{B}{B_0}$, where B is the mass of the marine boron reservoir and $B_0 = 5.628 \times 10^{17}$mol

$B_{carbonate}$	Boron deposition associated with marine carbonate burial	$B_{carbonate,0} = 5.55 \times 10^{9} molyr^{-1}$, $B_{carbonate} = B_{carbonate,0} \cdot \frac{mccb}{mccb_0} \cdot \frac{B}{B_0}$
$B_{crustlowTweath}$	Boron removal via low temperature crustal weathering	$B_{crustlowTweath,0} = 2.4977 \times 10^{10} molyr^{-1}$, $B_{crustlowTweath} = B_{crustlowTweath,0} \cdot \frac{B}{B_0}$

Supplementary Table 6: Relevant model parameters.

A	B	C	Source
16.88	4.2	0.13	[77]
16.0	4.14	0.13	[78]
15.35	4.23	0.14	[79]
13.81	4.58	0.08	[80]

Supplementary Table 7: Constants used during $T_{\delta^{18}O}$ calculations.

All data and the full model code are open accessed archived:

https://doi.pangaea.de/10.1594/PANGAEA.981213[81]

https://doi.org/10.5281/zenodo.15699150[82]

Supplementary References

1. Wilson, R. C. L., Hiscott, R. N., Willis, M. G. & Gradstein, F. M. "The Lusitanian Basin of west-central Portugal: Mesozoic and Tertiary tectonic, stratigraphic and subsidence history. Extensional Tectonics and Stratigraphy of the North Atlantic Margins" in *Extensional Tectonics and Stratigraphy of the North Atlantic Margins*, A. J. Tankard, H. R. Balkwill, Eds. (AAPG Memoir 1989), vol. 46.

2. Rasmussen, E. S., Lomholt, S., Andersen, C. & Vejbæk, O. V. Aspects of the structural evolution of the Lusitanian Basin in Portugal and the shelf and slope area offshore Portugal. *Tectonophysics* **300**, 199-225 (1998).

3. Alves, T. M., Moita, C., Cunha, T., Ullnaess, M., Myklebust, R., Monteiro, J. H., Manuppella, G. Diachronous evolution of Late Jurassic-Cretaceous continental rifting in the northeast Atlantic (west Iberian margin). *Tectonics* **28**, (2009).

4. Bjerrum, C. J., Surlyk, F., Callomon, J. H. & Slingerland, R. L. Numerical paleoceanographic study of the Early Jurassic Transcontinental Laurasian Seaway. *Paleoceanography* **16**, 390-404 (2001).

5. Bassoulet, J. P., Elmi, S., Poisson, A., Cecca, F., Bellion, Y., Guiraud, R. & Baudin, F. "Middle Toarcian (184-182 Ma)" in *Atlas Tethys Paleoenvironmental Maps*. J. Dercourt, L. E. Ricou, B. Vrielynck, Eds. (Gauthier-Villars, 1993), 63-80.

6. Gahr, M. E. Response of Lower Toarcian (Lower Jurassic) macrobenthos of the Iberian Peninsula to sea level changes and mass extinction. *J. Iber. Geol.* **31**, 197-215 (2005).

7. Duarte, L. V. Lithostratigraphy, sequence stratigraphy and depositional setting of the Pliensbachian and Toarcian series in the Lusitanian Basin (Portugal). *Ciências da Terra* **16**, 17-23 (2007).

8. Silva, R. L., Duarte, L. V. & Comas-Rengifo, M. J. "Facies and Carbon Isotope Chemostratigraphy of Lower Jurassic Carbonate Deposits, Lusitanian Basin (Portugal): Implications and Limitations to the Application in Sequence Stratigraphic Studies" in *Chemostratigraphy*, M, Ramkumar, Eds. (Elsevier, 2015), chap. 3, pp. 341-371.

9. Mouterde, R., Ruget-Perrot, Ch. & Almeida, F. M. Coupe du Lias au Sud de Condeixa. *Comun. Serv. Geol. Portugal* **48**, 61-91 (1964-1965).

10. Ferreira, J., Mattioli, E., Sucherás-Marx, B., Giraud, F., Duarte, L.V., Pittet, B., Suan, G., Hassler, A. & Spangenberg, J.E. Western Tethys Early and Middle Jurassic calcareous nannofossil biostratigraphy. *Earth-Sci. Rev.* **197**, 102908 (2019).

11. Comas-Rengifo, M. J., Duarte, L. V., García Joral, F. & Goy, A. Los braquiópodos del Toarciense Inferior (Jurásico) en el área de Rabaçal-Condeixa (Portugal): distribución estratigráfica y paleobiogeográfica. *Comunicações geológicas* **100**, Especial I, 37-42 (2013).

12. Correia, V. F., Riding, J. B., Duarte, L. V., Fernandes, P. & Pereira, Z. The Early Jurassic palynostratigraphy of the Lusitanian Basin, western Portugal. *Geobios* **51**, 537-557 (2018).

13. Duarte, L. V., Comas-Rengifo, M., García Joral, F., Goy, A., Miguez-Salas, O. & Rodríguez-Tovar, F. "The TOAE in the Western Iberian Margin and its context within the Lower Jurassic evolution of the Lusitanian Basin. Sedimentological and macroinvertebrate record across the Lower Toarcian in the Rabaçal area - Stop 2.2." in *Field Trip Guidebook: The Toarcian Oceanic Anoxic Event in the Western Iberian Margin and its context within the Lower Jurassic evolution of the Lusitanian Basin*. Ed. by L. V. Duarte and R. L. Silva (University of Coimbra, 2018), pp. 71-82.

14. Rodrigues, B., Duarte, L. V., Silva, R. L. & Mendonça Filho, J. G. Sedimentary organic matter and early Toarcian environmental changes in the Lusitanian Basin (Portugal). *Palaeogeogr. Palaeoclimatol. Palaeoecol.* **554**, 109781 (2020).

15. Piazza, V. Duarte, L. V., Renaudie J. & Aberhan, M. Reductions in body size of benthic macroinvertebrates as a precursor of the early Toarcian (Early Jurassic) extinction event in the Lusitanian Basin, Portugal. *Paleobiology* **45**, 296-316 (2019).

16. Gahr, M. E. Palökologie des Makrobenthos aus dem Unter-Toarc SW-Europas: Doctoral dissertation, Universität Würzburg. *Beringeria* **31**, 3-204, ISSN 0937-0242 (2002).

17. Rodríguez-Tovar, F. J., Miguez-Salas, O. & Duarte, L. V. Toarcian Oceanic Anoxic Event induced unusual behaviour and palaeobiological changes in *Thalassinoides* tracemakers. *Palaeogeogr. Palaeoclimatol. Palaeoecol*. **485**, 46-56 (2017).

18. De Vicente, G., Vegas, R., Muñoz-Martín, A., van Wees, J. D., Casas-Sáinz, A., Sopeña, A., Sánchez-Moya, Y., Arche, A., López-Gómez, J., Olaiz, A. & Fernández-Lozano, J. Oblique strain partitioning and transpression on an inverted rift: The Castilian Branch of the Iberian Chain. *Tectonophysics* **470**, 224-242 (2009).

19. Gómez, J. J. & Goy, A. Late Triassic and Early Jurassic palaeogeographic evolution and depositional cycles of the Western Tethys Iberian platform system (Eastern Spain). *Palaeogeogr. Palaeoclimatol. Palaeoecol*. **222**, 77-94, (2005).

20. Gómez, J. J. & Fernández-López, S. R. The Iberian Middle Jurassic carbonate-platform system: Synthesis of the palaeogeographic elements of its eastern margin (Spain). *Palaeogeogr. Palaeoclimatol. Palaeoecol*. **236**, 190-205 (2006).

21. Piazza, V., Ullmann, C. V. & Aberhan, M. Ocean warming affected faunal dynamics of benthic invertebrate assemblages across the Toarcian Oceanic Anoxic Event in the Iberian Basin (Spain). *PLoS ONE* **15,** e0242331 (2020).

22. Comas-Rengifo, M. J., Gómez, J. J., Goy, A., Arias, C. F., Bernad, J., García Joral, F., Herrero, C., Martínez, G. & Perilli, N. "The Toarcian in the Rambla del Salto (Sierra Palomera) section." in *1st Toarcian and 4th Aalenian working groups meeting. Field-Trip Iberian Range Guide-Book*, S. Ureta, Ed. (Universidad Complutense de Madrid, 1996), pp. 27-48.

23. Kullberg, J., Olóriz, F., Marques, B., Caetano, P. & Rocha, R. Flat pebble conglomerates: A local marker for Early Jurassic seismicity related to synrift tectonics in the Sesimbra area (Lusitanian Basin, Portugal). *Sediment. Geol.* **139**, 49-70 (2001).

24. Duarte, L. V. & Soares, A. F. Litostratigrafia das séries margo-calcárias do Jurássico Inferior da Bacia Lusitânica (Portugal). *Com. Instituto Geológico e Mineiro* **89**, 135-154 (2002).

25. Ullmann, C. V., Boyle, R., Duarte, L. V., Hesselbo, S. P., Kasemann, S. A., Klein, T., Lenton, T. M., Piazza, V. & Aberhan, M. Warm afterglow from the Toarcian Oceanic Anoxic Event drives the success of deep-adapted brachiopods. *Sci. Rep.* **10**, 6549 (2020).

26. Ferreira, J., Mattioli, E., Pittet, B., Cachão, M. & Spangenberg, J.E. Palaeoecological insights on Toarcian and lower Aalenian calcareous nannofossils from the Lusitanian Basin (Portugal). *Palaeogeogr. Palaeoclimatol. Palaeoecol.* **436**, 245-262 (2015).

27. Ogg, J. G., Ogg, G. M. & Gradstein, F. M. *A concise geologic time scale 2016* (Amsterdam, Netherlands, Elsevier, 2016).

28. Ohnemueller, F., Prave, A. R., Fallick, A. E. & Kasemann, S. A. Ocean acidification in the aftermath of the Marinoan glaciation. Geology 42, 1103-1106 (2014).

29. Clarkson, M. O., Kasemann, S. A., Wood, R. A., Lenton, T. M., Daines, S. J., Richoz, S., Ohnemueller, F., Meixner, A., Poulton, S. W. & Tipper, E. T. Ocean acidification and the Permo-Triassic mass extinction. Science 348, 229-232 (2015).

30. Dickson, A. G. Thermodynamics of the dissociation of boric acid in synthetic seawater from 273.15 to 318.15 K. *Deep Sea Res. Part A Oceanogr. Res. Pap.* **37**, 755-766 (1990).

31. Dera, G. & Donnadieu, Y. Modeling evidences for global warming, Arctic seawater freshening, and sluggish oceanic circulation during the Early Toarcian anoxic event. *Paleoceanography* **27**, PA2211 (2012).

32. Locarnini, R. A., Mishonov, V., Antonov, J. L., Boyer, T. P. & Garcia, H. E. "World Ocean Atlas 2005, Volume 1: Temperature" in *S. Levitus*, Ed. (NOAA Atlas NESDIS 61, U.S. Gov.Printing Office, Washington, D.C. 2006), 182 pp.

33. Them, T. R., Gill, B. C., Caruthers, A. H., Gröcke, R., Tulsky, E. T., Martindale, R. C., Poulton, T. P. & Smith, P. L. High-resolution carbon isotope records of the Toarcian Oceanic Anoxic Event (Early Jurassic) from North America and implications for the global drivers of the Toarcian carbon cycle. *Earth Planet. Sci. Lett.* **459**, 118-126 (2017).

34. Percival, L., Cohen, A. S., Davies, M. K., Dickson, A. J., Hesselbo, S. P., Jenkyns, H. C., Leng, M. J., Mather, T. A., Storm, M. S & Xu, W. Osmium isotope evidence for two pulses of increased continental weathering linked to Early Jurassic volcanism and climate change. *Geology* **44**, 759-762 (2016).

35. Brazier, J.-M., Suan, G., Tacail, T., Simon, L., Martin, J. E., Mattioli, E. & Balter, V. Calcium isotope evidence for dramatic increase of continental weathering during the Toarcian oceanic anoxic event (Early Jurassic). Earth Planet. Sci. Lett. **411**, 164-176 (2015).

36. Duarte, L. V. Facies analysis and sequential evolution of the Toarcian–Lower Aalenian series in the Lusitanian Basin (Portugal). *Com. Instituto Geológico e Mineiro* **83**, 65-94 (1997).

37. Hönisch, B., Ridgwell, A., Schmidt, D., Thomas, E., Gibbs, S., Sluijs, A., Zeebe, R., Kump, L., Martindale, R., Greene, S., Kiessling, W., Ries, J., Zachos, J. C., Royer, D., Barker, S., Marchitto, T., Moyer, R., Pelejero, C., Ziveri, P. & Williams, B. The Geological Record of Ocean Acidification. *Science* **335**, 1058-1063 (2012).

38. Kasemann, S. A., Pogge von Strandmann, P. A. E., Prave, A. R., Fallick, A. E., Elliott, T. & Hoffmann, K. H. Continental weathering following a Cryogenian glaciation: Evidence from calcium and magnesium isotopes. *Earth Planet. Sci. Lett.* **396**, 66-77 (2014).

39. Burgess, S. D., Bowring, S. A., Fleming, T. H. & Elliot, D. H. High-precision geochronology links the Ferrar large igneous province with early-Jurassic ocean anoxia and biotic crisis. *Earth Planet. Sci. Lett.* **415**, 90-99 (2015).

40. Percival, L., Witt, M., Mather, T. A., Hermoso, M., Jenkyns, H. C., Hesselbo, S. P., Al-Suwaidi, A. H., Storm, M. S., Xu, W. & Ruhl, M. Globally enhanced mercury deposition during the end-Pliensbachian extinction and Toarcian OAE: A link to the Karoo-Ferrar Large Igneous Province. *Earth Planet. Sci. Lett.* **428**, 267-280 (2015).

41. McElwain, J. C., Wade-Murphy, J. & Hesselbo, S. P. Changes in carbon dioxide during an oceanic anoxic event linked to intrusion into Gondwana coals. *Nature* **435**, 479-82 (2005).

42. Svensen, H., Corfu, F., Polteau, S., Hammer, Ø. & Planke, S. Rapid magma emplacement in the Karoo Large Igneous Province. *Earth Planet. Sci. Lett.* **325-326**, 1-9 (2012).

43. Elliot, D. H. Geological and tectonic evolution of the Transantarctic Mountains: A review. *Geol. Soc. Spec. Publ.* **381**, 7-35 (2013).

44. Kemp, D. B., Coe, A. L., Cohen, A. S. & Schwark, L. Astronomical pacing of methane release in the Early Jurassic period. *Nature* **437**, 396-399 (2005).

45. Hermoso, M., Minoletti, F., Le Callonnec, L., Jenkyns, H. C., Hesselbo, S. P., Rickaby, R. E. M., Renard, M., de Rafélis, M. & Emmanuel, L. Global and local forcing of Early Toarcian seawater chemistry: A comparative study of different paleoceanographic settings (Paris and Lusitanian basins). *Paleoceanography* **24**, PA4208 (2009).

46. Beerling, D. J. & Brentnall, S. J. Numerical evaluation of mechanisms driving Early Jurassic changes in global carbon cycling. *Geology* **35**, 247-250 (2007).

47. Dera, G. & Donnadieu, Y. Modeling evidences for global warming, Arctic seawater freshening, and sluggish oceanic circulation during the Early Toarcian anoxic event. *Paleoceanography* **27**, PA2211 (2012).

48. Suan, G., Mattioli, E., Pittet, B., Mailliot, S. & Lécuyer, C. Evidence for major environmental perturbation prior to and during the Toarcian (Early Jurassic) oceanic anoxic event from the Lusitanian Basin, Portugal. *Paleoceanography* **23**, PA1202 (2008).

49. Mattioli, E., Pittet, B., Petitpierre, L. & Mailliot, S. Dramatic decrease of pelagic carbonate production by nannoplankton across the Early Toarcian anoxic event (T-OAE). *Glob. Planet. Change* **65**, 134-145 (2009).

50. Ferreira, J., Mattioli, E., Pittet, B., Cachão, M. & Spangenberg, J.E. Palaeoecological insights on Toarcian and lower Aalenian calcareous nannofossils from the Lusitanian Basin (Portugal). *Palaeogeogr. Palaeoclimatol. Palaeoecol.* **436**, 245-262 (2015).

51. Mattioli, E., Pittet, B., Suan, G. & Mailliot, S. Calcareous nannoplankton changes across the early Toarcian oceanic anoxic event in the western Tethys. *Paleoceanography* **23**, PA3208 (2008).

52. Peti, L. & Thibault, N. Abundance and size changes in the calcareous nannofossil Schizosphaerella – Relation to sea-level, the carbonate factory and palaeoenvironmental change from the Sinemurian to earliest Toarcian of the Paris Basin. *Palaeogeogr. Palaeoclimatol. Palaeoecol.* **485**, 271-282 (2017).

53. Correia, V. F., Riding, J. B., Fernandes, P., Duarte, L. V. & Pereira, Z. The palynology of the lower and middle Toarcian (Lower Jurassic) in the northern Lusitanian Basin, western Portugal. *Rev. Palaeobot. Palynol.* **237**, 75-879 (2017a).

54. Correia, V. F., Riding, J. B., Fernandes, P., Duarte, L. V. & Pereira, Z. The palynological response to the Toarcian Oceanic Anoxic Event (Early Jurassic) at Peniche, Lusitanian Basin, western Portugal. *Mar. Micropaleontol*. **137**, 46-63, (2017b).

55. Van de Schootbrugge, B., Houben, A., Ercan, F., Verreussel, R., Kerstholt, S., Janssen, N. M. M., Nikitenko, B. & Suan, G. Enhanced Arctic-Tethys connectivity ended the Toarcian Oceanic Anoxic Event in NW Europe. *Geol. Mag*. **157**, 1593-1611, (2020).

56. Rae, J. W. B., Foster, G. L., Schmidt, D. N. & Elliott, T. Boron isotopes and B/Ca in benthic foraminifera: Proxies for the deep ocean carbonate system. *Earth and Planetary Science Letters* **302**, 403-413 (2011).

57. Levi, A., Müller, W., Erez, J. Intrashell variability of trace elements in benthic foraminifera grown under high CO_2 levels. *Front. Earth Sci.* **7**. doi: 10.3389/feart.2019.00247 (2019).

58. Donald, H.K, Ries, J.B., Stewart, J.A., Fowell, S.E, Foster, G.L. Boron isotope sensitivity to seawater pH change in a species of Neogoniolithon coralline red alga. *Geochimica et Cosmochimica Acta*. **217**, 240-253 (2017)

59. Piazza, G., Paredes, E., Bracchi, V. A., Pena, L. D., Hall-Spencer, J. M., Ferrara, C., Cacho, I., Basso, D. Multi-specific calibration of the B isotope proxy in calcareous red algae for pH reconstruction, EGU General Assembly 2023, Vienna, Austria, 24–28 Apr 2023, EGU23-12561, https://doi.org/10.5194/egusphere-egu23-12561, 2023.

60. Cornwall, C. E., Comeau, S., McCulloch, M.T. Coralline algae elevate pH at the site of calcification under ocean acidification. *Glob Change Biol.* **23**, 4245-4256 (2017).

61. Liu, Y.-W., Rokitta, S.D., Rost, B., Eagle, R.A. Constraints on coccolithophores under ocean acidification obtained from boron and carbon geochemical approaches. *Geochimica et Cosmochimica Acta*. 315, 317-332 (2021).

62. Paris, G., Bartolini, A., Donnadieu, Y., Beaumont, V. & Gaillardet, J. Investigating boron isotopes in a middle Jurassic micritic sequence: Primary vs. diagenetic signal. *Chem. Geol.* **275**, 117-126 (2010).

63. Kasemann, S. A., Prave, A. R., Fallick, A. E., Hawkesworth, C. J. & Hoffmann, K.-H. Neoproterozoic ice ages, boron isotopes, and ocean acidification: Implications for a snowball Earth. *Geology* **38**, 775-778 (2010).

64. Lenton, T. M., Daines, S. J. & Mills, B. J. W. COPSE Reloaded: An improved model of biogeochemical cycling over Phanerozoic time. Earth Sci. Rev. 178, 1-28 (2018).

65. Tostevin, R. & Mills, J. W. Reconciling proxy records and models of Earth's oxygenation during the Neoproterozoic and Palaeozoic. *Interface Focus* **10**, 2019037 (2020).

66. Lemarchand, D., Gaillardet, J., Lewin, E. & Allegre, C. J. Boron isotope systematics in large rivers: Implications for the marine boron budget and paleo pH reconstruction over the Cenozoic. *Chem. Geol*. **190**, 123-140 (2002).

67. Klochko, K., Kaufman, A. J., Yao, W., Byrne, R. H. & Tossell, J. A. Experimental measurement of boron isotope fractionation in seawater. *Earth Planet. Sci. Lett.* **248**, 276-285 (2006).

68. Jurikova, H., Liebetrau, V., Gutjahr, M., Rollion-Bard, C., Hu, M. Y., Krause, S., Henkel, D., Hiebenthal, C., Schmidt, M., Laudien, J. & Eisenhauer, A. Boron isotope systematics of cultured brachiopods: Response to acidification, vital effects and implications for palaeo-pH reconstruction. *Geochim. Cosmochim. Acta* **248**, 370-386 (2019).

69. Lécuyer, C., Grandjean, P., Reynard, B., Albarède, F. & Telouk, P. 11B/10B analysis of geological materials by ICP MS Plasma 54: Application to the boron fractionation between brachiopod calcite and seawater. *Chem. Geol.* **186**, 45-55, (2002).

70. Penman, D. E., Hönisch, B., Rasbury, E. T., Hemming, N. G. & Spero, H. J. Boron, carbon, and oxygen isotopic composition of brachiopod shells: Intra-shell variability, controls, and potential as a paleo-pH recorder. *Chem. Geol.* **340**, 32-39 (2013).

71. Hansen, J., Sato, M., Russell, G. & Kharecha, P. Climate sensitivity, sea level and atmospheric carbon dioxide. Philos. *Trans. R. Soc. A Math. Phys. Eng. Sci.* **371**, 20120294 (2013).

72. Brand, U., Azmy, K., Bitner, M. A., Logan, A., Zuschin, M., Came, R. & Ruggiero, E. Oxygen isotopes and MgCO3 in brachiopod calcite and a new paleotemperature equation. Chem. Geol. 359, 23-31 (2013).

73. Marshall, J. D., Pirrie, D., Clarke, A., Nolan, C. P. & Sharman, J. Stable-isotopic composition of skeletal carbonates from living Antarctic marine invertebrates. *Lethaia* **29**, 203-212 (1997).

74. Page, K. N. The Lower Jurassic of Europe: its subdivision and correlation. *GEUS Bulletin* **1**, 21-59 (2003).

75. Cervantes, P. & Wallace, P. J. Role of H_2O in subduction-zone magmatism: New insights from melt inclusions in high-Mg basalts from central Mexico. *Geology* **31**, 235-238 (2003).

76. Svensen, H., Planke, S., Chevallier, L., Malthe-Sørenssen, A., Corfu, F. & Jamtveit, B. Hydrothermal venting of greenhouse gases triggering Early Jurassic global warming. *Earth Planet. Sci. Lett.* **3-4**, 554-566 (2007).

77. Craig, H. "The measurement of oxygen isotope paleotemperatures" in *Stable Isotopes in Oceanographic Studies and Paleotemperatures*, E. Tongiorgi, Ed. (Consiglio Nazionale delle Ricerche Laboratorio di Geologia Nucleare, Pisa, 1965), pp. 161-182.

78. Anderson, T.F. & Arthur, M.A. "Stable isotopes of oxygen and carbon and their application to sedimentologic and paleoenvironmental problems" in *Stable Isotopes in Sedimentary Geology*, M. A. Arthur, T. F. Anderson, I. R. Kaplan, J. Veizer, L. S. Land, Eds. (SEPM Society for Sedimentary Geology, 1983), vol. 10, pp. 1-151.

79. Epstein, S. & Mayeda, T. K. Variation of O^{18} Content of Waters from Natural Sources. *Geochim. Cosmochim. Acta* **4**, 213-224 (1953).

80. Leng, M. J. & Marshall, J. D. Palaeoclimate interpretation of stable isotope data from lake sediment archives. *Quat. Sci. Rev.* **23**, 811-831 (2004).

81. Kasemann, S. A., Klein, T., Boyle, R. A, Ullmann, C. V., Aberhan, M., Meixner, A., Duarte, L. V., Lenton, T. M., Piazza, V. & Wood, R. A. Boron, carbon and oxygen isotope data from micrite, brachiopods and bivalves from the Early Jurassic Toarcian Oceanic Anoxic Event in SW Europe [dataset bundled publication]. PANGAEA, https://doi.pangaea.de/10.1594/PANGAEA.981213 (2025).

82. Boyle, R. Ocean acidification at the Toarcian Anoxic Event captured by boron isotopes in the lime mud record. Zenodo. https://doi.org/10.5281/zenodo.15699150 (2025).
