# Supplementary material for: Ocean acidification at the Toarcian Anoxic Event captured by boron isotopes in the lime mud record
Source: Commun Earth Environ. 2025 Jul 5;6(1):533. doi: 10.1038/s43247-025-02510-y (PMC12228580; doi:10.1038/s43247-025-02510-y)
Supplement: Supplementary file 3 — Reporting Summary [file 43247_2025_2510_MOESM3_ESM.pdf]

nature portfolio

Corresponding author(s): Simone Kasemann

Last updated by author(s): Jun 14, 2025

Reporting Summary

Nature Portfolio wishes to improve the reproducibility of the work that we publish. This form provides structure for consistency and transparency in reporting. For further information on Nature Portfolio policies, see our Editorial Policies and the Editorial Policy Checklist.

Statistics

For all statistical analyses, confirm that the following items are present in the figure legend, table legend, main text, or Methods section.

n/a	Confirmed	
☐	☒	The exact sample size (*n*) for each experimental group/condition, given as a discrete number and unit of measurement
☐	☒	A statement on whether measurements were taken from distinct samples or whether the same sample was measured repeatedly
☐	☒	The statistical test(s) used AND whether they are one- or two-sided<br>*Only common tests should be described solely by name; describe more complex techniques in the Methods section.*
☐	☒	A description of all covariates tested
☒	☐	A description of any assumptions or corrections, such as tests of normality and adjustment for multiple comparisons
☐	☒	A full description of the statistical parameters including central tendency (e.g. means) or other basic estimates (e.g. regression coefficient) AND variation (e.g. standard deviation) or associated estimates of uncertainty (e.g. confidence intervals)
☐	☒	For null hypothesis testing, the test statistic (e.g. *F*, *t*, *r*) with confidence intervals, effect sizes, degrees of freedom and *P* value noted<br>*Give P values as exact values whenever suitable.*
☒	☐	For Bayesian analysis, information on the choice of priors and Markov chain Monte Carlo settings
☒	☐	For hierarchical and complex designs, identification of the appropriate level for tests and full reporting of outcomes
☒	☐	Estimates of effect sizes (e.g. Cohen's *d*, Pearson's *r*), indicating how they were calculated

Our web collection on statistics for biologists contains articles on many of the points above.

Software and code

Policy information about availability of computer code

Data collection	Full model code is available at: https://github.com/richboyle111/Boron/blob/main/COPSE%20Boron.zip.
Data analysis	Full model code is available at: https://github.com/richboyle111/Boron/blob/main/COPSE%20Boron.zip.

For manuscripts utilizing custom algorithms or software that are central to the research but not yet described in published literature, software must be made available to editors and reviewers. We strongly encourage code deposition in a community repository (e.g. GitHub). See the Nature Portfolio guidelines for submitting code & software for further information.

Data

Policy information about availability of data

All manuscripts must include a data availability statement. This statement should provide the following information, where applicable:

- Accession codes, unique identifiers, or web links for publicly available datasets
- A description of any restrictions on data availability
- For clinical datasets or third party data, please ensure that the statement adheres to our policy

All data are open accessed archived in the World Data Center PANGAEA at: https://doi.pangaea.de/10.1594/PANGAEA.981213

Research involving human participants, their data, or biological material

Policy information about studies with human participants or human data. See also policy information about sex, gender (identity/presentation), and sexual orientation and race, ethnicity and racism.

Reporting on sex and gender	NA
Reporting on race, ethnicity, or other socially relevant groupings	NA
Population characteristics	NA
Recruitment	NA
Ethics oversight	NA

Note that full information on the approval of the study protocol must also be provided in the manuscript.

Field-specific reporting

Please select the one below that is the best fit for your research. If you are not sure, read the appropriate sections before making your selection.

☐ Life sciences ☐ Behavioural & social sciences ☒ Ecological, evolutionary & environmental sciences

For a reference copy of the document with all sections, see nature.com/documents/nr-reporting-summary-flat.pdf

Ecological, evolutionary & environmental sciences study design

All studies must disclose on these points even when the disclosure is negative.

Study description	Boron, carbon and oxygen isotope study of carbonate rocks (micrite), calcitic rhynchonellid brachiopods and calcitic bivalves (oyster) to trace ocean pH changes.
Research sample	Samples include three components of the carbonate rock record - micrite (lime mud), calcitic rhynchonellid brachiopods (brachiopod order Rhynchonellida: Choffatirhynchia, Cirpa, Gibbirhynchia, Homoeorhynchia, Quadratirhynchia and Soaresirhynchia) and the dominantly calcitic bivalve (oyster) Gryphaea (bivalve order Ostreida). Preservation evaluation, element concentrations, and oxygen and carbon isotopes are published in Ullmann et al. Sci. Rep. 10, 6549 (2020).
Sampling strategy	Samples were collected at distances varying from 0.1 to 11 m scale intervals, covering the upper Pliensbachian to the middle Toarcian, including the Pliensbachian – Toarcian boundary and the Toarcian Oceanic Anoxic Event.
Data collection	Macroscopic (laboratory), microscopic (scanning electron microscope) and geochemical (carbon and oxygen isotopes, trace elements) data were collected at the University of Exeter and the University of Bremen. .
Timing and spatial scale	The samples were taken in the field in 2016 and 2017 and the analysis data was collected between 2017 and 2020.
Data exclusions	Macroscopically screened samples with obvious signs of secondary alteration and meteoric diagenesis were excluded.
Reproducibility	As in general, the samples were geochemically analysed at least three times; if the sample quantity permitted, the samples were fully treated and analysed at least twice. The accuracy and repeatability of the geochemical analyses were checked using various reference materials.
Randomization	Samples were grouped into (1) micrite (lime mud), (2) calcitic rhynchonellid brachiopods and (3) bivalves
Blinding	The samples were analysed with a different ID than the actual sample number.

Did the study involve field work? ☒ Yes ☐ No

Field work, collection and transport

Field conditions	The natural vegetation consists of bushes and meadows, it was dry and the daytime temperatures were around 25 degrees.
Location	Rabaçal/Fonte Coberta, Portugal; 40°03'08.0"N 8°27'30.5"W and Barranco de la Cañada, Spain; 40°23'53.4"N 1°30'07.4"W

Access & import/export	Fossil material from Fonte Coberta / Rabaçal is archived at the Museum für Naturkunde, Berlin, Germany (samples MB.B.10843-10912 for brachiopods and MB.B.20325-20346 for bivalves). Shell fragments from Barranco de la Cañada are stored at the Museu de Ciencias Naturales, Zaragoza, Spain (samples MPZ 2019/415-571)
Disturbance	Small samples (about 100 mg of carbonate whole rock) were taken from natural outcrops where no visible disturbance occurred.

Reporting for specific materials, systems and methods

We require information from authors about some types of materials, experimental systems and methods used in many studies. Here, indicate whether each material, system or method listed is relevant to your study. If you are not sure if a list item applies to your research, read the appropriate section before selecting a response.

Materials & experimental systems

n/a	Involved in the study
☒	☐ Antibodies
☒	☐ Eukaryotic cell lines
☒	☐ Palaeontology and archaeology
☒	☐ Animals and other organisms
☒	☐ Clinical data
☒	☐ Dual use research of concern
☒	☐ Plants

Methods

n/a	Involved in the study
☒	☐ ChIP-seq
☒	☐ Flow cytometry
☒	☐ MRI-based neuroimaging

Plants

Seed stocks	NA
Novel plant genotypes	NA
Authentication	NA
